# The GAPDH redox switch safeguards reductive capacity and enables survival of stressed tumour cells

**DOI:** 10.1038/s42255-023-00781-3

**Published:** 2023-04-06

**Authors:** Deepti Talwar, Colin G. Miller, Justus Grossmann, Lukasz Szyrwiel, Torsten Schwecke, Vadim Demichev, Ana-Matea Mikecin Drazic, Anand Mayakonda, Pavlo Lutsik, Carmen Veith, Michael D. Milsom, Karin Müller-Decker, Michael Mülleder, Markus Ralser, Tobias P. Dick

**Affiliations:** 1 Division of Redox Regulation, DKFZ-ZMBH Alliance, German Cancer Research Center (DKFZ), Heidelberg, Germany; 2 Department of Biochemistry, Charité—Universitätsmedizin Berlin, Corporate Member of Freie Universität Berlin and Humboldt-Universität zu Berlin, Berlin, Germany; 3 Division of Experimental Hematology, German Cancer Research Center (DKFZ), Heidelberg, Germany; 4 Heidelberg Institute for Stem Cell Technology and Experimental Medicine (HI-STEM), Heidelberg, Germany; 5 Division of Cancer Epigenomics, German Cancer Research Center (DKFZ), Heidelberg, Germany; 6 Laboratory of Computational Cancer Biology and Epigenomics, Department of Oncology, Catholic University (KU) Leuven, Leuven, Belgium; 7 Core Facility Tumor Models, German Cancer Research Center (DKFZ), Heidelberg, Germany; 8 Core Facility High Throughput Mass Spectrometry, Charité—Universitätsmedizin Berlin, Corporate Member of Freie Universität Berlin and Humboldt-Universität zu Berlin, Berlin, Germany; 9 The Wellcome Centre for Human Genetics, Nuffield Department of Medicine, University of Oxford, Oxford, UK; 10 Faculty of Biosciences, Heidelberg University, Heidelberg, Germany

**Keywords:** Metabolism, Proteins, Cancer, Cell biology

## Abstract

Glyceraldehyde 3-phosphate dehydrogenase (GAPDH) is known to contain an active-site cysteine residue undergoing oxidation in response to hydrogen peroxide, leading to rapid inactivation of the enzyme. Here we show that human and mouse cells expressing a GAPDH mutant lacking this redox switch retain catalytic activity but are unable to stimulate the oxidative pentose phosphate pathway and enhance their reductive capacity. Specifically, we find that anchorage-independent growth of cells and spheroids is limited by an elevation of endogenous peroxide levels and is largely dependent on a functional GAPDH redox switch. Likewise, tumour growth in vivo is limited by peroxide stress and suppressed when the GAPDH redox switch is disabled in tumour cells. The induction of additional intratumoural oxidative stress by chemo- or radiotherapy synergized with the deactivation of the GAPDH redox switch. Mice lacking the GAPDH redox switch exhibit altered fatty acid metabolism in kidney and heart, apparently in compensation for the lack of the redox switch. Together, our findings demonstrate the physiological and pathophysiological relevance of oxidative GAPDH inactivation in mammals.

## Abstract (web summary)

Human and mouse cells expressing a redox-insensitive GAPDH mutant that otherwise retains its catalytic activity are shown to be unable to upregulate the oxidative pentose phosphate pathway in response to oxidative stress.

## Main

The glycolytic enzyme glyceraldehyde 3-phosphate dehydrogenase (GAPDH) is unusual in that it is readily oxidized at its active site cysteine in response to moderate elevations of intracellular hydrogen peroxide (H_2_O_2_) levels^1,2^. No other enzyme is as conspicuously oxidized by H_2_O_2_ as GAPDH^2,3^, although several other metabolic enzymes, including pyruvate kinase^4^, are also known to be sensitive to oxidation. Indeed, GAPDH stands out against other thiol-containing proteins because the H_2_O_2_ reactivity of its active site thiol is higher than is typical of protein thiols in general^5^. GAPDH thiol oxidation first leads to sulfenylation (R-SH + H_2_O_2_ → R-SOH + H_2_O), and then to glutathionylation (R-SOH + GSH → R-SSG + H_2_O). The result is the reversible inhibition of GAPDH glycolytic activity^6^.

H_2_O_2_-mediated GAPDH oxidation was recognized more than 50 years ago^7^, but initially it was widely assumed that GAPDH oxidation represents an inadvertent side reaction, potentially reflecting protein damage. Only some 15 years ago, it became clear that oxidative GAPDH inactivation benefits oxidant-exposed cells rather than being deleterious. Yeast cells exposed to H_2_O_2_ reroute glucose flux from glycolysis into the oxidative branch of the pentose phosphate pathway (oxPPP), thus increasing the re-generation of reducing equivalents in the form of NADPH. As a result, these cells become H_2_O_2_ tolerant^8,9^. In these experiments, activation of the oxPPP was found to correlate with oxidative GAPDH inactivation and a computational metabolic model based on ordinary differential equations suggested the possibility that GAPDH oxidation may be the cause of oxPPP activation^8^. Subsequently, oxidative-stress-induced oxPPP activation was also observed in mammalian cells^10^, but the biological relevance of GAPDH oxidation remained unclear. Indeed, a causal connection between GAPDH oxidation and oxPPP activation has not been demonstrated in any mammalian system. H_2_O_2_ may be a common cause of both GAPDH oxidation and oxPPP activation, thus explaining the observed correlation. Along these lines, two recent studies have proposed that oxPPP activation may be independent of GAPDH oxidation and instead depend on the de-inhibition of glucose-6-phosphate dehydrogenase (G6PDH), to unlock ‘reserve flux capacity’ of the oxPPP, at least in non-yeast species^11,12^. Thus, it has remained an open question whether oxidative inactivation of GAPDH, de-inhibition of G6PDH or the combination of both, is necessary and sufficient for oxPPP activation. If GAPDH oxidation is indeed relevant to mammalian cells, a related as yet unanswered question is the physiological context and function of GAPDH oxidation in mammals. Under which physiological or pathological circumstances does an elevation of endogenous H_2_O_2_ activate the GAPDH redox switch? Or, in other words, what would be the consequence for mammalian cells if they expressed a non-oxidizable GAPDH instead of the oxidizable one?

So far, it has been a challenge to prove cause and causality, because it is not obvious how oxidizability of GAPDH can be abolished without also compromising its glycolytic activity. Mutating the active site cysteine is not an option, as this will not only prevent oxidation but also glycolytic activity. However, the situation changed when the molecular mechanism of GAPDH oxidation was uncovered^5^. H_2_O_2_ reactivity of GAPDH was found to depend on a dedicated H_2_O_2_ binding site (adjacent to but separate from the glyceraldehyde 3-phosphate (G3P) binding site) and on a dedicated mechanism that supports the transition state of the reaction (R-SH + H_2_O_2_ → R-SOH + H_2_O) as well as leaving group departure (that is, the hydroxide ion is protonated to leave as a water molecule). By mutating one of several amino acid residues involved in this mechanism (including Tyr-314 and Thr-177 in human GAPDH), selective ablation of oxidation sensitivity was achieved, that is, without compromising normal GAPDH activity. This showed that separate mechanisms mediate the reactivity of the same thiolate towards either H_2_O_2_ or G3P. Thus, GAPDH is one of the few known redox-regulated proteins that can be oxidized by means of a ‘built-in’ H_2_O_2_ binding and reaction site, and in that sense is comparable to the prototypical H_2_O_2_ stress sensor from bacteria, OxyR^13^.

On the basis of these insights and considerations, this work used isogenic pairs of mammalian cell lines, engineered by clustered regularly interspaced short palindromic repeats (CRISPR)-based genome editing to differ by a single GAPDH amino acid, to explore the biological role and relevance of GAPDH oxidation sensitivity. The mutations used are highly conservative, minimizing potential structural perturbance: the Y314F mutation effectively removes a single hydroxyl group from the enzyme, and the T177A mutation (corresponding to T175A in mouse GAPDH) removes a hydroxyl together with a methyl group.

In this Article, we present three major findings. First, we demonstrate causality, namely that GAPDH oxidation is required for rapid oxPPP activation, NADPH regeneration and oxidative stress tolerance in mammalian cells. We find that G6PDH activation (de-inhibition), the mechanism believed to access the reserve flux capacity of the oxPPP, is insufficient for adequate oxPPP activation in the absence of GAPDH oxidation. Second, we show that the GAPDH redox switch is functionally relevant in the context of tumour biology, as it supports anchorage-independent growth of single cells and spheroids. Cells lacking the GAPDH redox switch are unfit to cope with the increase of endogenous peroxide stress that is triggered by the loss of anchorage. Similarly, tumour growth in vivo is accompanied by peroxide stress and is strongly suppressed when the GAPDH switch is disabled in tumour cells. Pro-oxidative chemo- and radiotherapy synergized with the deactivation of the GAPDH switch, potentially suggesting novel strategies for tumour combination therapy. Third, we show that the GAPDH switch impacts metabolism under physiological conditions in the context of the whole organism, suggesting a role in regulating reductive capacity even under non-stress conditions.

## Results

### The glycolysis‒oxPPP switch depends on GAPDH oxidation

**Fig. 1:**
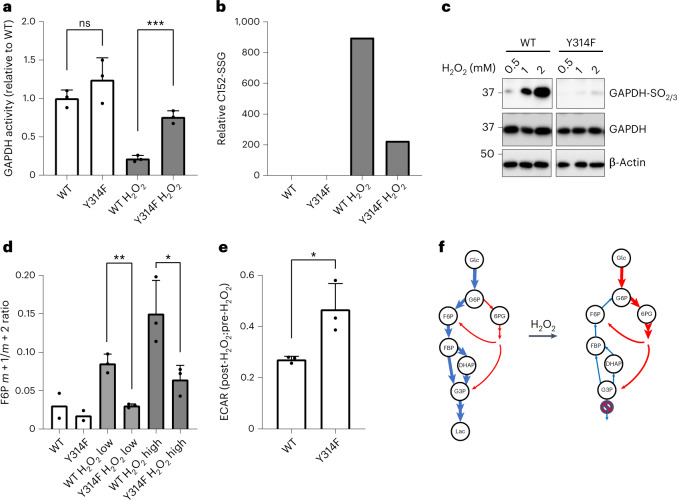
The glycolysis‒oxPPP switch depends on GAPDH oxidation. **a**, GAPDH activity as measured in the lysate of HAP1 cells expressing either WT or mutant (Y314F) GAPDH, and following treatment of intact cells with 100 µM H_2_O_2_ for 5 min. Activity values are normalized relative to untreated WT cells. Data are presented as mean ± standard deviation (s.d.), based on *n* = 3 biological replicates with *n* = 3 technical replicates each. NS, non-significant; ***P < 0.001; *P* = 0.2413 and 0.0005, based on a two-tailed unpaired *t*-test. **b**, Glutathionylation of GAPDH Cys-152 in WT and mutant cells, before and after treatment of intact cells with 50 µM H_2_O_2_ for 5 min, as determined by LC–MS/MS analysis. Bars represent the mean of *n* = 3 technical replicates. **c**, Hyperoxidation (sulfinylation and/or sulfonylation) of GAPDH Cys-152 as visualized by immunoblotting before and after treatment of intact cells with up to 2 mM H_2_O_2_ for 5 min. Representative of *n* = 3 independent experiments. **d**, Partitioning of [1,2-^13^C]glucose flux into glycolysis and PPP, as determined by the isotopic signature of F6P. WT and mutant cells were left untreated, or treated with either 10 µM (low) or 50 µM (high) H_2_O_2_ for 30 s. Data are presented as mean ± s.d., based on *n* = 3 for H_2_O_2_ treated samples. *P < 0.05, **P < 0.01; *P* = 0.0016 and 0.0347, based on a two-tailed unpaired *t*-test. **e**, Change in the extracellular acidification rate (ECAR) in response to treatment of WT and mutant cells with 100 µM H_2_O_2_. Data are presented as mean ± s.d. (*n* = 3 biological replicates with *n* = 3 technical replicates each). *P < 0.05; *P* = 0.0291, based on a two-tailed unpaired *t*-test. **f**, Scheme depicting the rerouting of glucose flux between glycolysis and PPP in response to GAPDH oxidation. Blue arrows: glycolytic flux; red arrows: PPP flux. Please note that F6P and G3P are intermediates of both glycolysis and the PPP. Hence, GAPDH oxidation allows for the cycling of intermediates between the PPP and upper glycolysis, as indicated by reversed arrows in upper glycolysis (right). Source data

**Extended Data Fig. 1:**
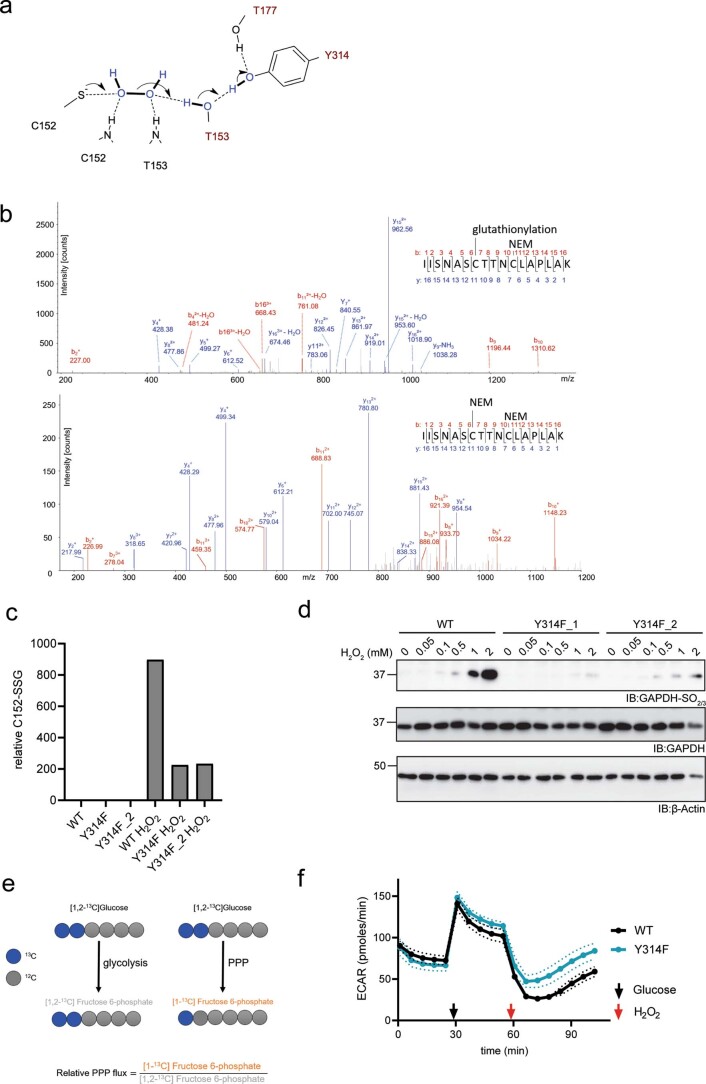
Mutational inactivation of GAPDH oxidation sensitivity. (**a**) Previously suggested proton relay mechanism facilitating GAPDH oxidation by H_2_O_2_. Cys-152 (C152) is the active site cysteine. The hydroxyl group of Tyr-314 (Y314) is required for the protonation of the hydroxide leaving group. The hydroxyl group of Thr-177 (T177) is thought to support the proper positioning of the Tyr-314 hydroxyl group. See Peralta et al., 2015, for further details. Dotted lines: hydrogen bonds. (**b**) Representative LC-MS/MS spectra of the GAPDH tryptic peptide containing the active site cysteine, before (lower panel) and after (upper panel) cellular H_2_O_2_ exposure, demonstrating selective S-glutathionylation of the active site cysteine under oxidative stress conditions. The calculated mass for fragment y11^2+^ of the glutathionylated peptide is 782.8 Da, the calculated mass of the corresponding alkylated fragment y11^2+^ is 701.8 Da. (**c**) Glutathionylation of GAPDH Cys-152 in WT and mutant cells, before and after treatment of intact cells with 50 µM H_2_O_2_ for 5 min, as determined by LC-MS/MS analysis. Same experiment as shown in main Fig. 1b, but including a second independently selected clone of the mutant cell line (Y314F_2). Bars represent the mean of 3 technical replicates. Representative of n = 3 biological replicates. (**d**) Hyperoxidation (sulfinylation and/or sulfonylation) of GAPDH Cys-152 as visualized by immunoblotting before and after treatment of intact cells with up to 2 mM H_2_O_2_ for 5 min. Same experiment as shown in main Fig. 1c, but including additional H_2_O_2_ concentrations and a second independently selected clone of the mutant cell line (Y314F_2). Representative of n = 3 biological replicates. (**e**) Outline of the strategy to determine relative flux of [1,2-^13^C]glucose into glycolysis and the pentose phosphate pathway (PPP), respectively. The ratio of singly and doubly labeled fructose-6-phosphate is taken to indicate relative PPP flux. (**f**) Extracellular acidification rate (ECAR) of cells expressing either WT or mutant GAPDH in response to 10 mM glucose (black arrow) and 100 µM H_2_O_2_ (red arrow). Based on n = 6 biological replicates. Dotted lines: SD. Source data

To evaluate the role of GAPDH oxidation sensitivity in a whole-cell context, we edited the genome of human HAP1 cells. Considering the mechanism facilitating GAPDH oxidation sensitivity^5^ (Extended Data Fig. 1a), we rendered GAPDH oxidation-insensitive by introducing the Y314F point mutation into the GAPDH genomic locus. Two mutant cell clones were independently selected for comparison. In agreement with previous in vitro and yeast experiments^5^, basal GAPDH activity did not differ significantly between wild type (WT) and mutant (Y314F) cells. However, GAPDH activity in mutant cells was less sensitive to inhibition by peroxide (Fig. 1a). Indeed, when intact cells were exposed to H_2_O_2_, mutant GAPDH exhibited less *S*-glutathionylation at its active site cysteine (Fig. 1b and Extended Data Fig. 1b), which was also less susceptible to hyperoxidation (Fig. 1c). The two independently generated mutant cell lines exhibited a similar insensitivity to GAPDH oxidation, relative to WT cells (Extended Data Fig. 1c,d). We then asked how the mutation affects metabolic flow through glycolysis and the PPP. To this end, we performed metabolic isotopologue labelling experiments with [1,2-^13^C] glucose to differentiate between fructose 6-phosphate (F6P) generated by glycolysis (retaining both ^13^C atoms), and F6P generated by the PPP (retaining only one ^13^C atom), hence providing a relative measure of glucose 6-phosphate (G6P) flux into the two alternative metabolic branches (Extended Data Fig. 1e)^14^. The ratio between doubly and singly labelled F6P (measured after 30 s) revealed an immediate increase of PPP flux in WT cells, which was significantly diminished in mutant cells (Fig. 1d). In line with the differences in relative PPP flux, H_2_O_2_ suppressed lactate generation more strongly in WT versus mutant cells (Fig. 1e and Extended Data Fig. 1f). Taken together, these findings supported the notion that oxidative GAPDH inactivation is a requirement for H_2_O_2_-dependent glycolysis-to-PPP rerouting (Fig. 1f).

### Oxidative GAPDH inactivation safeguards reductive capacity

**Fig. 2:**
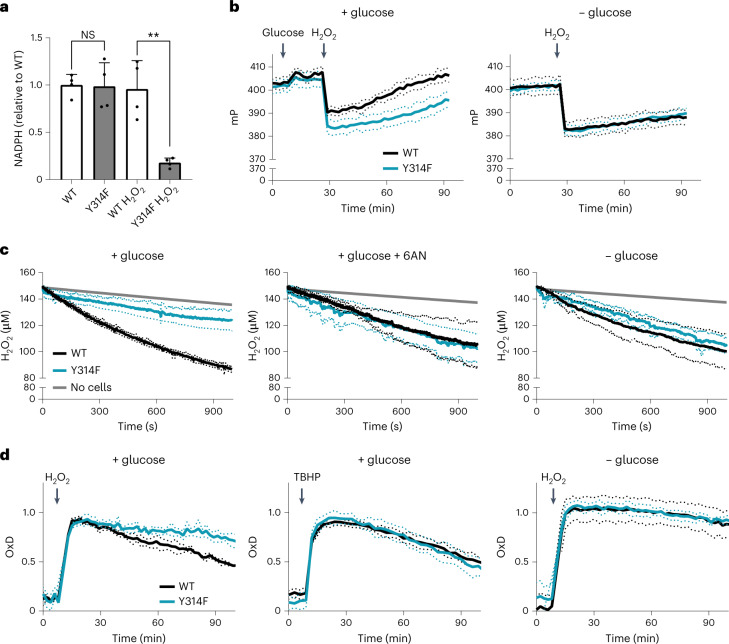
Oxidative GAPDH inactivation safeguards reductive capacity. **a**, Relative NADPH levels as measured in the cell lysate of WT and mutant (Y314F) HAP1 cells, before and after treatment of intact cells with 100 µM H_2_O_2_ for 3 min. Bars represent the mean of *n* = 3 biological replicates ± standard deviation (s.d.). NS, non-significant; **P < 0.01; *P* = 0.9206 and 0.0022, based on a two-tailed unpaired *t*-test. **b**, NADP^+^-dependent G6PDH dimerization triggered by 100 µM H_2_O_2_, as measured by the fluorescence polarization response of the Apollo-NADP^+^ probe, in the presence (left) or absence (right) of 10 mM glucose. mP: millipolarization units. Based on the mean of *n* = 3 biological replicates. Dotted lines: s.d. **c**, Loss of extracellular H_2_O_2_ (starting concentration: 150 µM) from the cell culture supernatant as measured with an H_2_O_2_-selective electrode, in the presence (left and middle) or absence (right) of 10 mM glucose, and in the presence of 10 mM 6-aminonicotinamide (6-AN) (middle). Based on *n* = 3 biological replicates. Dotted lines: s.d. **d**, Cytoplasmic H_2_O_2_ levels in response to exogenous application of 100 µM H_2_O_2_ (left and right) or 50 µM TBHP (middle) as measured by the roGFP2-Orp1 probe, in the presence (left and middle) or absence (right) of 10 mM glucose. Based on the mean of *n* = 3 biological replicates with *n* = 3 technical replicates each. Dotted lines: s.d. Source data

The above findings suggested that cells with an intact GAPDH redox switch should be better able to maintain NADPH levels under oxidative stress conditions relative to cells lacking the switch. Indeed, cells expressing WT GAPDH, but not cells expressing oxidation-insensitive GAPDH, were able to maintain NADPH levels upon H_2_O_2_ exposure (Fig. 2a). To further evaluate the impact of the GAPDH mutation on endogenous NADPH regeneration, we monitored NADP^+^-dependent dimerization of the glucose-6-phosphate dehydrogenase (G6PDH) domains in the fluorescence anisotropy probe ‘Apollo-NADP^+^’^15^. In mutant cells, H_2_O_2_ induced a larger drop in fluorescence polarization, reflecting G6PDH dimerization, indicating their limited capacity to acutely regenerate NADPH from NADP^+^ (Fig. 2b, left). In the absence of glucose, the fluorescence polarization difference was abolished (Fig. 2b, right). Consistently, cells with WT (that is, oxidizable) GAPDH removed H_2_O_2_ from the medium more rapidly than cells with mutant GAPDH (Fig. 2c, left). Pharmacological inhibition of the oxPPP with 6-aminonicotinamide^16^ abolished this difference (Fig. 2c, middle), as did the withdrawal of glucose (Fig. 2c, right). The genetically encoded H_2_O_2_ probe roGFP2-Orp1 confirmed that the intracellular reductive capacity is indeed compromised in mutant cells relative to WT cells (Fig. 2d, left). There was no difference in reductive capacity when cells metabolize *tert*-butyl hydroperoxide (TBHP) instead of H_2_O_2_ (Fig. 2d, middle), as expected, because TBHP is not a substrate for the GAPDH oxidation mechanism^5^. Likewise, there was no difference in reductive capacity in the absence of glucose (Fig. 2d, right). Together, these results showed that oxidative inactivation of GAPDH is causally connected to increased influx of glucose into the oxPPP, increased NADPH regeneration and the safeguarding of reductive capacity in mammalian cells.

### GAPDH(T175A) in MEFs mimics GAPDH(Y314F) in HAP1 cells

**Extended Data Fig. 2:**
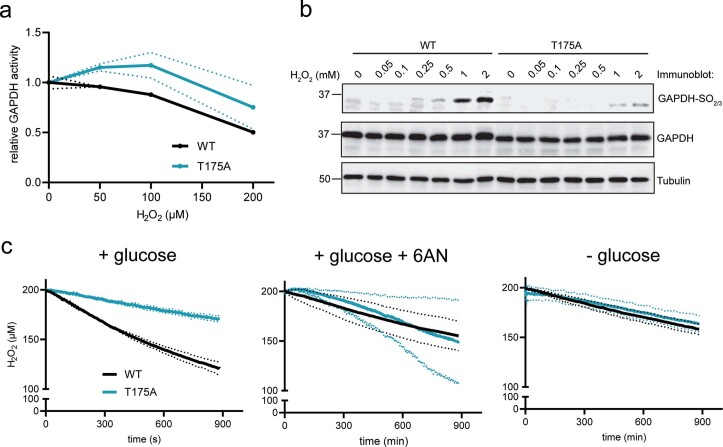
GAPDH(T175A) in MEFs mimics GAPDH(Y314F) in HAP1 cells. (**a**) GAPDH activity as measured in the lysate of MEF cells expressing either WT or mutant (T175A) GAPDH, following treatment of intact cells with 200 µM H_2_O_2_ for 5 min. Activity values are normalized relative to untreated WT cells. Based on n = 3 biological replicates. Dotted lines: SD. (**b**) Hyperoxidation (sulfinylation and/or sulfonylation) of GAPDH Cys-152 in WT and mutant (T175A) MEF cells as visualized by immunoblotting before and after treatment of intact cells with up to 2 mM H_2_O_2_ for 5 min. Representative of n = 3 biological replicates. (**c**) Depletion of extracellular H_2_O_2_ (starting concentration: 200 µM) from the cell culture supernatant as measured with an H_2_O_2_-selective electrode, in the presence (left and middle panels) or absence (right panel) of 10 mM glucose, and in the presence of 10 mM 6-aminonicotinamide (6AN) (middle panel). Based on n = 3 biological replicates. Dotted lines: SD. Source data

To further substantiate that changes in metabolism, reductive capacity and oxidative stress resistance are caused by the loss of GAPDH oxidation sensitivity, we analysed an alternative GAPDH point mutation in the context of genome-edited mouse embryonic fibroblasts (MEFs). The T175A mutation (corresponding to T177A in human GAPDH) is functionally analogous to the Y314F mutation, in that it has been found to cause oxidation-insensitivity^5^. Measuring GAPDH activity in MEF lysates, there was no detectable difference in basal activity, but mutant GAPDH(T175A) was more resistant to H_2_O_2_-induced inactivation (Extended Data Fig. 2a) and hyperoxidation (Extended Data Fig. 2b). Like human cells expressing GAPDH(Y314F), murine cells expressing GAPDH(T175A) were slower than corresponding WT cells in removing H_2_O_2_ from the supernatant (Extended Data Fig. 2c, left) and the difference was abolished by blocking the PPP or withdrawing glucose (Extended Data Fig. 2c, middle and right). In conclusion, two different GAPDH mutations (created with different guide RNAs in different mammalian cell types) lead to similar effects, strongly supporting the conclusion that GAPDH oxidation insensitivity per se is the cause of the observed changes.

### GAPDH oxidation supports proliferation under peroxide stress

**Fig. 3:**
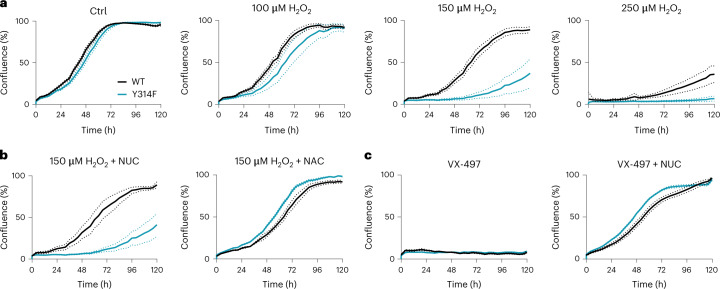
GAPDH oxidation supports proliferation under peroxide stress. **a**, Cell growth, as measured by optical imaging, of WT and mutant cells, either left untreated (left), or seeded in the presence of 100, 150 or 250 µM H_2_O_2_ (middle and right). Based on *n* = 5 technical replicates, representative of *n* = 3 biological replicates. Dotted lines: standard deviation (s.d.). **b**, Cell growth, as measured by optical imaging, of WT and mutant cells seeded in the presence of 150 µM H_2_O_2_, and then treated with 30 µM of nucleotide (NUC) mixture (left) or 10 mM of NAC (right). Based on *n* = 5 technical replicates, representative of *n* = 3 biological replicates. Dotted lines: s.d. **c**, Cell growth, as measured by optical imaging, of WT and mutant cells seeded with 5 µM of the IMPDH inhibitor merimepodib (VX-497), in the absence (left) or presence (right) of the nucleotide (NUC) mixture (30 µM). Based on *n* = 5 technical replicates, representative of *n* = 3 biological replicates. Dotted lines: s.d. Source data

**Extended Data Fig. 3:**
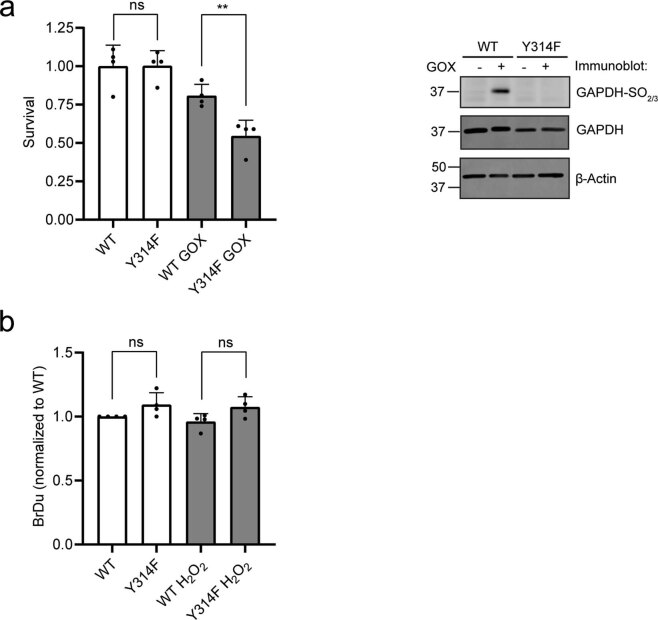
The GAPDH redox switch allows cells to grow under peroxide stress. (**a**) Survival of WT and mutant HAP1 cells after 24 h of exposure to low levels of H_2_O_2_ continuously produced by glucose oxidase (GOX) (left panel). Data are presented as mean values ± SD, based on n = 4 biological replicates. ns: non-significant; **p < 0.01; p = 0.9774 and 0.0062, based on a two-tailed unpaired t-test. Visualization of GAPDH hyperoxidation after 24 h in the same experiment (right panel). Representative of n = 4 biological replicates. (**b**) DNA synthesis, as measured by BrdU incorporation, in WT and mutant HAP1 cells, in the absence (bars 1-2) or presence (bars 3-4) of 100 µM H_2_O_2_. Data are presented as mean values ± SD, based on n = 4 biological replicates with n = 5 technical replicates each. ns: non-significant; p = 0.0952 and 0.0651, based on a two-tailed unpaired t-test. Source data

We then asked how the GAPDH redox switch affects cellular growth under oxidative stress conditions. We seeded cells in the presence or absence of H_2_O_2_ and monitored their proliferation by optical imaging. When seeded in the absence of H_2_O_2_, WT and mutant cells grew similarly (Fig. 3a, left). However, when seeded in the presence of a single bolus of H_2_O_2_, WT cells had a significant survival and growth advantage (Fig. 3a, centre and right). Cells expressing oxidation-resistant GAPDH also had a significant survival disadvantage under conditions of continuous low-level peroxide exposure (Extended Data Fig. 3a). We then asked if H_2_O_2_-induced growth inhibition of mutant cells can be rescued by providing either nucleotides or reducing equivalents, considering that both are products of the PPP. The suppression of mutant cell growth by H_2_O_2_ was not rescued by a nucleotide mixture (Fig. 3b, left), but almost fully rescued by *N*-acetyl cysteine (NAC) (Fig. 3b, right). The same nucleotide mixture reversed the growth-suppressing effect of the IMPDH inhibitor merimepodib (VX-497) in both WT and mutant cells, demonstrating its activity (Fig. 3c). In line with these results, WT and mutant cells exhibited similar rates of DNA synthesis (Extended Data Fig. 3b). Taken together, these results suggested that the GAPDH redox switch is critical when cells encounter oxidative stress during the phase of substrate attachment. Moreover, the resulting growth defect appears to be caused by a lack of PPP-derived reducing equivalents rather than a lack of PPP-derived nucleotide precursors.

### The GAPDH switch allows cells to survive the loss of anchorage

**Fig. 4:**
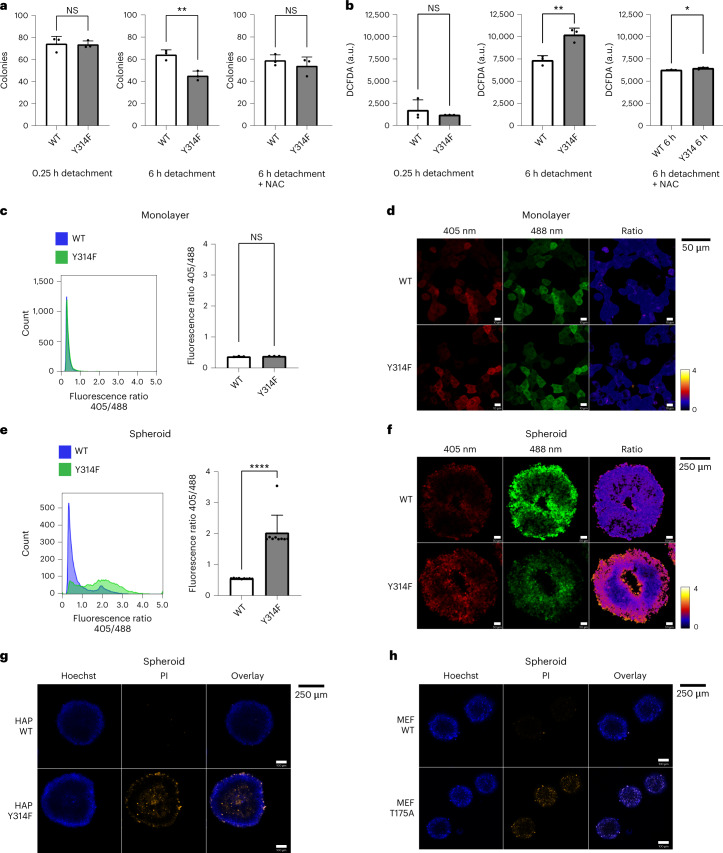
The GAPDH switch allows cells to survive the loss of anchorage. **a**, Colonies formed after seeding of 100 WT or mutant (Y314F) HAP1 cells into adherent cell culture plates. Before seeding, singularized cells were kept in ultralow-attachment plates for either 0.25 h (left), or 6 h (middle and right), in the absence (left and middle) or presence (right) of 10 mM NAC. Data are presented as mean ± standard deviation (s.d.), based on *n* = 3 biological replicates with *n* = 3 technical replicates each. NS, non-significant; **P < 0.01; *P* = 0.8860, 0.0059 and 0.4174, based on a two-tailed unpaired *t*-test. **b**, Relative intracellular oxidant levels as indicated by DCF fluorescence, corresponding to **a**. Data are presented as mean ± s.d., based on *n* = 3 biological replicates with *n* = 3 technical replicates each. NS, non-significant; *P < 0.05; **P < 0.01; *P* = 0.4696, 0.0057 and 0.0418, based on a two-tailed unpaired *t*-test. **c**, The roGFP2-Orp1 fluorescence ratio in WT and mutant (Y314F) HAP1 cells grown in adherent monolayer cell culture. Left: representative histogram. Right: quantitation presented as mean ± s.d. based on *n* = 3 biological replicates. NS, non-significant; *P* = 0.1583, based on a two-tailed unpaired *t*-test. **d**, Visualization of roGFP2-Orp1 in WT (top) and mutant (bottom) HAP1 cells grown in adherent monolayer culture. Fluorescence was recorded following sequential excitation at 405 nm (left) and 488 nm (middle). The fluorescence ratio image (right) was calculated pixel-by-pixel and colour-coded. Representative of *n* = 3 biological replicates. **e**, The roGFP2-Orp1 fluorescence ratio in WT and mutant (Y314F) HAP1 cells grown in non-adherent spheroid culture. Left: representative histogram. Right: quantitation presented as mean ± s.d., based on *n* = 9 biological replicates. ****P < 0.0001, based on a two-tailed unpaired *t*-test. **f**, Visualization of roGFP2-Orp1 in WT (top) and mutant (bottom) HAP1 cells grown in non-adherent spheroid culture. Fluorescence was recorded following sequential excitation at 405 (left) and 488 nm (middle). The fluorescence ratio image (right) was calculated pixel-by-pixel and colour-coded. Representative of *n* = 6 (WT) and *n* = 7 (Y314F) biological replicates. **g**, Staining of WT (top) and mutant (bottom) HAP1 spheroids with Hoechst dye (left) or PI (middle). Representative of *n* = 3 biological replicates. **h**, Staining of WT (top) and mutant (bottom) EF spheroids with Hoechst dye (left) or PI (middle). Representative of *n* = 3 biological replicates. Source data

**Extended Data Fig. 4:**
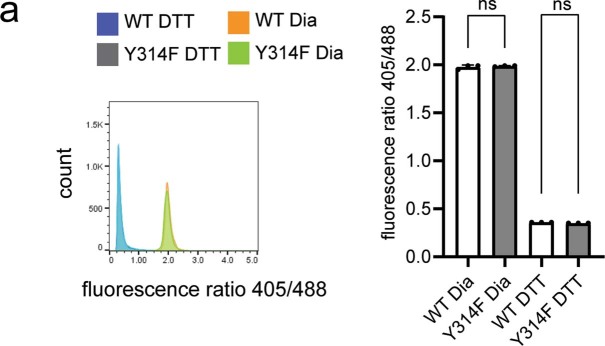
H_2_O_2_ levels associated with cell attachment. (**a**) The roGFP2-Orp1 405 nm/488 nm fluorescence ratio as measured by flow cytometry in WT and mutant (Y314F) HAP1 cells grown in adherent monolayer culture, following treatment with either 20 mM diamide (Dia) (full oxidation) or 100 mM dithiothreitol (DTT) (full reduction). Left panel: representative histogram. Right panel: Data are presented as mean values ± SD, based n = 3 biological replicates (unpaired two-tailed t test). ns: non-significant, based on a two-tailed unpaired t-test. Source data

Although there are situations in which mammalian cells must withstand exogenously produced peroxides, for example, at inflammatory sites, in other conditions a cellular response to endogenously produced peroxides is required. One such condition is the detachment of cells from the matrix and/or other cells. It has been observed repeatedly that detachment and/or low cell density elevates endogenous oxidant levels^17–19^. To investigate if the GAPDH redox switch is important under such conditions, we detached cells from the cell culture plastic surface, kept them detached and then determined their colony forming potential. Following a short detachment period (15 min), WT and mutant cells formed colonies with the same efficiency and did not show a difference in their endogenous oxidant levels (Fig. 4a,b, left). However, following a longer detachment period (6 h), mutant cells exhibited decreased clonogenic capacity and increased oxidant levels relative to WT cells (Fig. 4a,b, middle). These differences were abated by treatment with NAC (Fig. 4a,b, right). Next, we studied the 3D growth of WT and mutant cells as spheroids. It has been observed previously that growth inside a spheroid elevates endogenous oxidative stress^18,20^. In monolayer culture, both WT and mutant cells exhibited the same low levels of endogenous H_2_O_2_ (Fig. 4c,d and Extended Data Fig. 4a). In 3D growth, despite comparable spheroid size, mutant spheroids were more oxidized (Fig. 4e,f) and contained more dead cells (Fig. 4g) than WT spheroids. The same observation was made with WT and mutant MEFs under conditions of spheroid growth (Fig. 4h). Taken together, these results suggested that oxidative GAPDH inactivation protects mammalian cells against the elevation of endogenous peroxide levels that occurs in the context of anchorage-independent growth.

### The GAPDH redox switch protects tumour cells in vivo

**Fig. 5:**
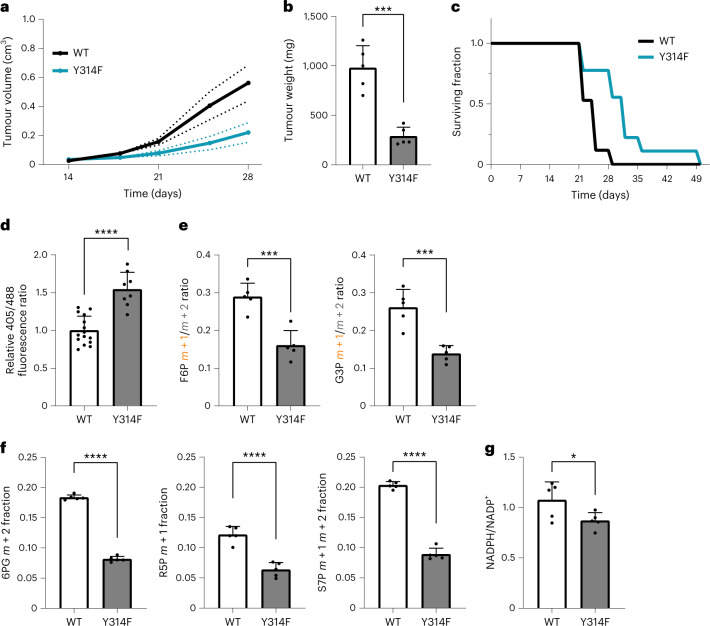
The GAPDH redox switch protects tumour cells in vivo. **a**, Growth of xenograft tumours after subcutaneous injection of HAP1 cells expressing either WT or mutant (Y314F) GAPDH. Based on *n* = 6 mice per group. Solid lines represent the mean, dotted lines represent the standard error of the mean. **b**, Weight of tumours explanted 28 days after xenografting. Data are presented as mean ± standard deviation (s.d.), based on *n* = 5 mice per group. ***P < 0.001; *P* = 0.0002, based on a two-tailed unpaired *t*-test. **c**, Survival time of tumour-bearing mice until reaching humane endpoints. Based on *n* = 17 (WT) and *n* = 9 (Y314F) mice. **d**, The roGFP2-Orp1 405 nm/488 nm fluorescence ratio as measured in cells isolated from explanted WT and mutant tumours. Data are presented as mean ± s.d., based on *n* = 15 (WT) and *n* = 8 (Y314F) mice. ****P < 0.0001, based on a two-tailed unpaired *t*-test. **e**, Metabolic flux analysis following tail vein injection of [1,2-^13^C]glucose and subsequent tumour explantation. The partitioning of glucose flux into glycolysis and PPP is indicated by the ratio of singly and doubly labelled F6P (left) and G3P (right). Data are presented as mean ± s.d, based on *n* = 5 mice per group. ***P < 0.001; *P* = 0.0007 and 0.0008, based on a two-tailed unpaired *t*-test. **f**, Metabolic flux analysis following tail vein injection of [1,2-^13^C]glucose and subsequent tumour explantation. The influx of [1,2-^13^C]glucose into the PPP is indicated by mass shifts in the PPP intermediates 6PG (left), R5P (middle) and S7P (right). Data are presented as mean ± s.d., based on *n* = 5 mice per group. ****P < 0.0001, based on a two-tailed unpaired *t*-test. **g**, NADPH/NADP^+^ ratio in WT and mutant tumours as determined by mass spectrometry. Data are presented as mean ± s.d., based on *n* = 5 mice per group. *P < 0.05; *P* = 0.0488, based on a two-tailed unpaired *t*-test. Source data

Tumour cells experience episodes of oxidative stress when growing as tumours in situ^21,22^. We therefore asked if tumour cells exploit the GAPDH redox switch to survive and grow inside the host environment. We transplanted WT and mutant HAP1 cells under the skin of NOD SCID gamma mice and monitored their growth. Tumours harbouring oxidation-resistant GAPDH grew significantly slower (Fig. 5a,b), suggesting that the loss of the GAPDH redox switch was a disadvantage for the tumour. Correlating with slower tumour growth, the survival time of animals bearing mutant tumours was longer (Fig. 5c). Analysing tumours grown from HAP1 cells expressing the cytosolic roGFP2-Orp1 H_2_O_2_ probe, we found mutant tumours to exhibit more probe oxidation than WT tumours (Fig. 5d). Performing metabolic flux analysis by injecting [1,2-^13^C]glucose into the tail vein, we determined that mutant tumours had lower PPP flux than WT tumours (Fig. 5e), which was also reflected by decreased labelling of key PPP intermediates (Fig. 5f) and a lower NADPH/NADP^+^ ratio (Fig. 5g). Together, these results suggested that tumour cells lacking the GAPDH redox switch are less well equipped to counteract oxidative stress and hence have a lower probability of survival in the host environment.

### Chemo- and radiotherapy synergize with deficient GAPDH oxidation

**Fig. 6:**
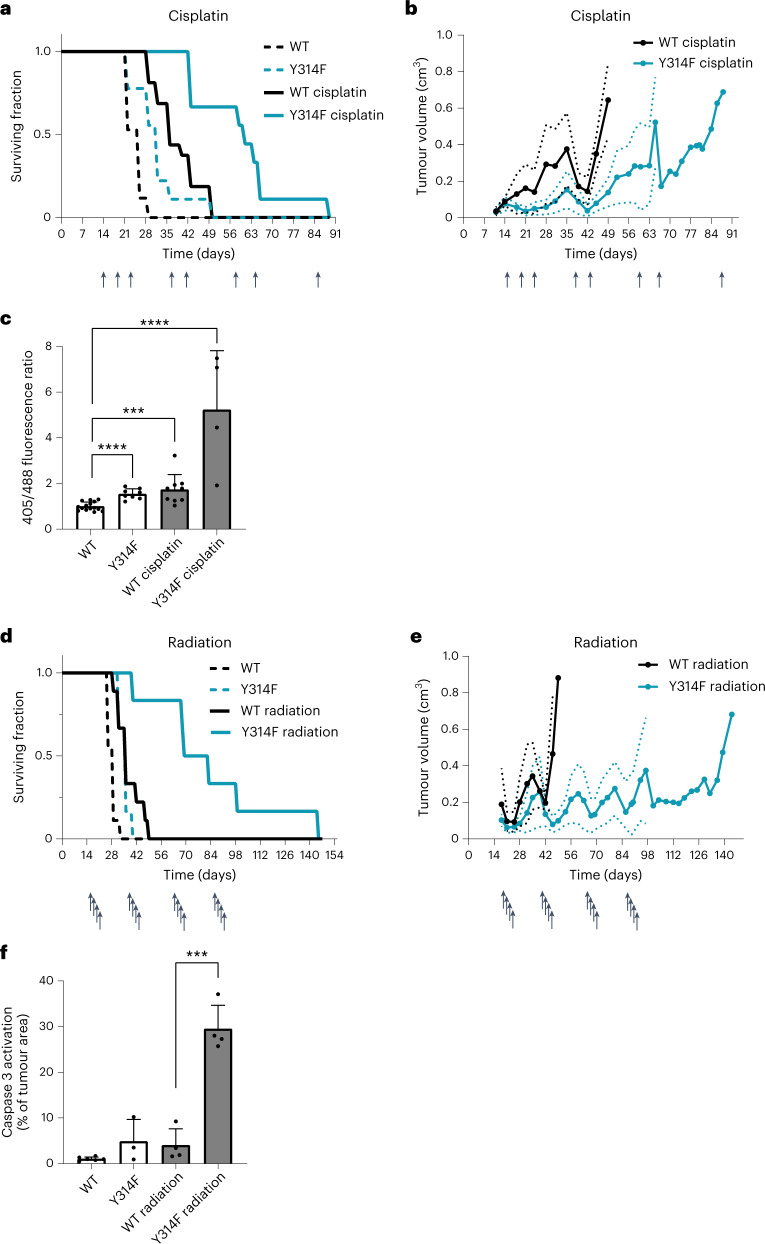
Chemo- and radiotherapy synergize with GAPDH oxidation insensitivity. **a**, Survival of tumour-bearing mice in response to cisplatin treatment, based on tumour size (>1.3 cm in any dimension) as the sole endpoint. Arrows indicate timepoints of treatment. Based on *n* = 17 (WT), *n* = 9 (Y314F), *n* = 16 (WT cisplatin) and *n* = 9 (Y314F cisplatin) mice. **b**, Growth kinetics of tumours expressing WT or mutant GAPDH, either mock-treated or treated with cisplatin. Arrows indicate timepoints of treatment. Based on *n* = 16 (WT cisplatin) and *n* = 9 (Y314F cisplatin) mice. **c**, The roGFP2-Orp1 405 nm/488 nm fluorescence ratio as measured in cells isolated from explanted WT and mutant tumours, either mock-treated or treated with cisplatin. Data are presented as mean ± standard deviation (s.d.), based on *n* = 15 (WT), *n* = 8 (Y314F), *n* = 9 (WT cisplatin) and *n* = 4 (Y314F cisplatin) mice. ***P < 0.001; ****P < 0.0001; *P* = <0.0001, 0.0004 and <0.0001, based on a two-tailed unpaired *t*-test. **d**, Survival of tumour-bearing mice either mock-treated or treated with ionizing radiation. Arrows indicate cycles of fractionated radiation. Based on *n* = 9 (WT), *n* = 6 (Y314F), *n* = 9 (WT radiation) and *n* = 6 (Y314F radiation) mice. **e**, Growth kinetics of tumours expressing WT and mutant GAPDH, untreated or treated with ionizing radiation. Arrows indicate cycles of fractionated radiation. Based on *n* = 9 (WT radiation) and *n* = 6 (Y314F radiation) mice. **f**, Quantitation of activated Caspase 3 in slices of tumours expressing WT or mutant GAPDH, untreated or treated with ionizing radiation. Data are presented as mean ± s.d., based on *n* = 6 (WT), *n* = 3 (Y314F), *n* = 4 (WT radiation) and *n* = 4 (Y314F radiation) mice. ***P < 0.001; *P* = 0.0002, based on a two-tailed unpaired *t*-test. Source data

**Extended Data Fig. 5:**
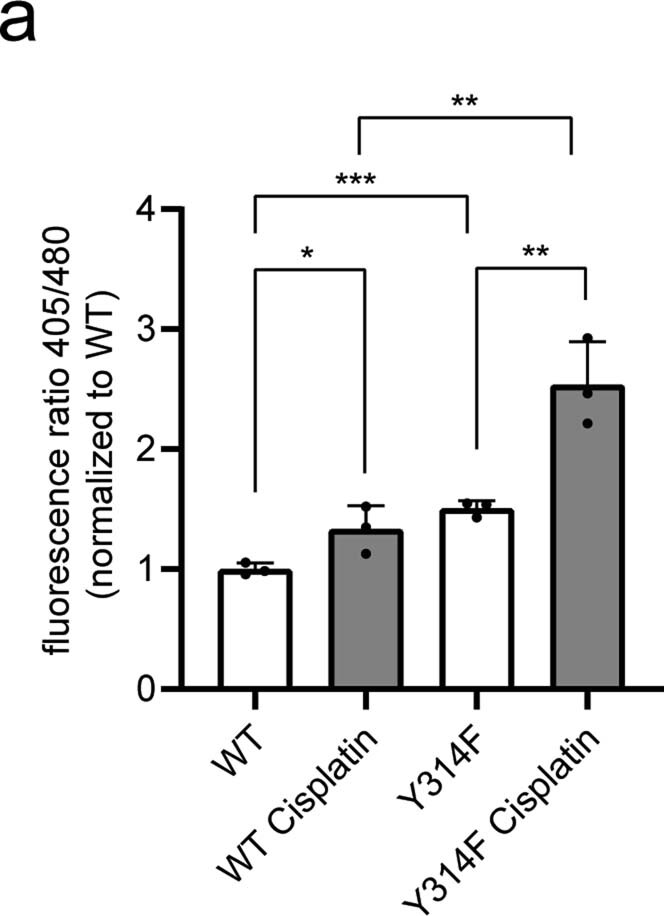
Cisplatin induces an intracellular elevation of H_2_O_2_ levels. (**a**) The roGFP2-Orp1 405 nm/488 nm fluorescence ratio as measured by flow cytometry of WT and mutant (Y314F) HAP1 cells, stably expressing the roGFP2-Orp1 probe, either mock-treated (bars 1, 3), or treated with 40 µM Cisplatin (bars 2, 4) for 24 h. Data are presented as mean values ± SD, based on n = 3 biological replicates with n = 4 technical replicates each. *p < 0.05; **p < 0.01; ***p < 0.01; p = 0.0477, 0.0004, 0.0072 and 0.0083, based on a two-tailed unpaired t-test. Source data

Several kinds of chemotherapy elevate oxidative stress in tumour cells^23^. We therefore asked if loss of the GAPDH redox switch would make tumour cells more responsive to chemotherapy. Cisplatin treatment is well known to trigger pro-oxidative effects^24^. Cell culture experiments confirmed that cisplatin elevates endogenous H_2_O_2_ levels, more strongly in mutant than in WT HAP1 cells (Extended Data Fig. 5a). While cisplatin treatment prolonged the survival of all tumour-bearing animals, it synergized with GAPDH oxidation insensitivity, as the survival benefit was more pronounced in animals with mutant tumours (Fig. 6a). Moreover, the re-growth of mutant tumours between treatment cycles was significantly delayed relative to WT tumours (Fig. 6b). Analysing tumours expressing the cytosolic roGFP2-Orp1 H_2_O_2_ probe, we observed that cisplatin elevated peroxide levels much more strongly in mutant than in WT tumours (Fig. 6c). We then asked if treatment with ionizing radiation would also synergize with GAPDH oxidation insensitivity. Indeed, while fractionated ionizing radiation prolonged the survival of all tumour-bearing animals, it caused a much more pronounced survival benefit in animals with mutant tumours (Fig. 6d) and strongly delayed the re-growth of mutant tumours between treatment cycles (Fig. 6e). The synergy between GAPDH oxidation insensitivity and radiation treatment was also reflected by intratumoural caspase-3 activation (Fig. 6f). Together, these observations showed that the GAPDH redox switch allows tumours to counteract oxidative stress that is elevated by either chemo- or radiotherapeutic treatment in vivo.

### A defective GAPDH switch alters metabolism in various tissues

**Fig. 7:**
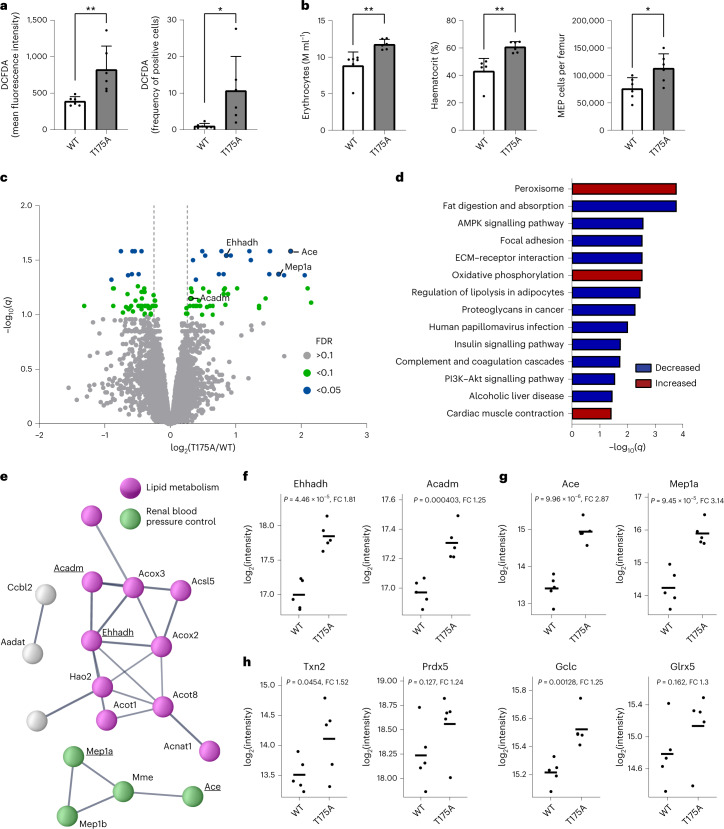
A defective GAPDH switch alters metabolism in various tissues. **a**, Relative intracellular oxidant levels in erythrocytes isolated from WT and mutant (T175A) animals. DCF fluorescence (left) and frequency of DCF-positive cells (right). Data are presented as mean ± standard deviation (s.d.), based on *n* = 6 mice per group. *P < 0.05; **P < 0.01; *P* = 0.0091 and 0.0301, based on a two-tailed unpaired *t*-test. **b**, Numbers of erythrocytes per millilitre (left), haematocrit (middle) and number of MEP cells per femur (right) of WT and mutant (T175A) animals. Data are presented as mean ± s.d., based on *n* = 6 mice per group. *P < 0.05; **P < 0.01; *P* = 0.0049, 0.0016 and 0.0208, based on a two-tailed unpaired *t*-test. **c**, Proteins differentially expressed in GAPDH mutant kidneys. Grey, yellow and orange dots represent proteins with a false discovery rate (FDR) >10%, <10% and <5%, respectively. Vertical lines indicate fold change thresholds of ±1.25. Based on *n* = 5 mice per group. **d**, Gene set enrichment analysis of proteins differentially expressed in the GAPDH mutant kidney. Mapping to KEGG pathways. Blue bars: gene sets with decreased representation in mutant kidneys. Red bars: gene sets with increased representation in mutant kidneys. Based on *n* = 5 mice per group. ECM, extracellular matrix. **e**, Interaction analysis of proteins most strongly upregulated in the GAPDH mutant kidney. Edges represent experimentally supported interactions (confidence score >0.7) from the STRING database (https://string-db.org). Purple nodes represent proteins included in Uniprot pathway ‘Lipid metabolism’ (KW-0443), and green nodes represent proteins with a specific function in blood pressure control. **f**, Examples of enzymes involved in β-oxidation. Based on *n* = 5 mice per group. FC, fold change. **g**, Examples of enzymes involved in blood pressure control. Based on *n* = 5 mice per group. **h**, Examples of redox enzymes upregulated in the GAPDH mutant kidney. Based on *n* = 5 mice per group. Source data

**Extended Data Fig. 6:**
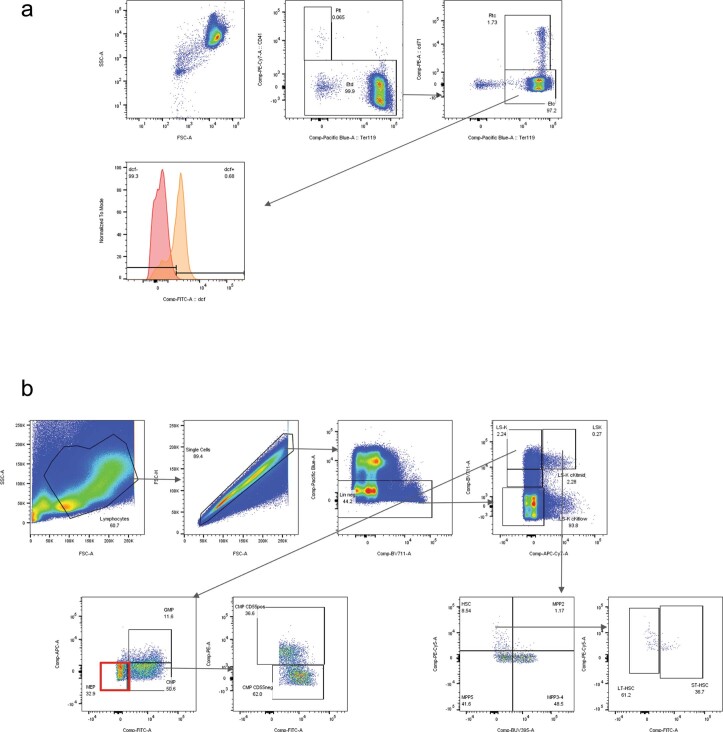
FACS Gating Strategies. (**a**) Gating scheme for flow cytometry-based analysis of ROS levels in erythrocytes from peripheral blood (Fig. 7a). Sequential flow cytometry gates were used to define: CD41- Ter119+ erythroid cells; CD71-, Ter119+ erythrocytes. The level of fluorescence of the DCFDA stain was assessed for this cell population within the FITC channel of the flow cytometer. (**b**) Gating scheme for flow cytometry-based identification of MEP cells from bone marrow (Fig. 7b). Sequential flow cytometry gates were used to define: live leukocytes based on forward scatter versus side scatter; single cells based on forward scatter area versus forward scatter height; lineage negative cells; c-Kit+, Sca-1- cells; CD34-, CD16/32- MEPs (indicated by red box).

**Extended Data Fig. 7:**
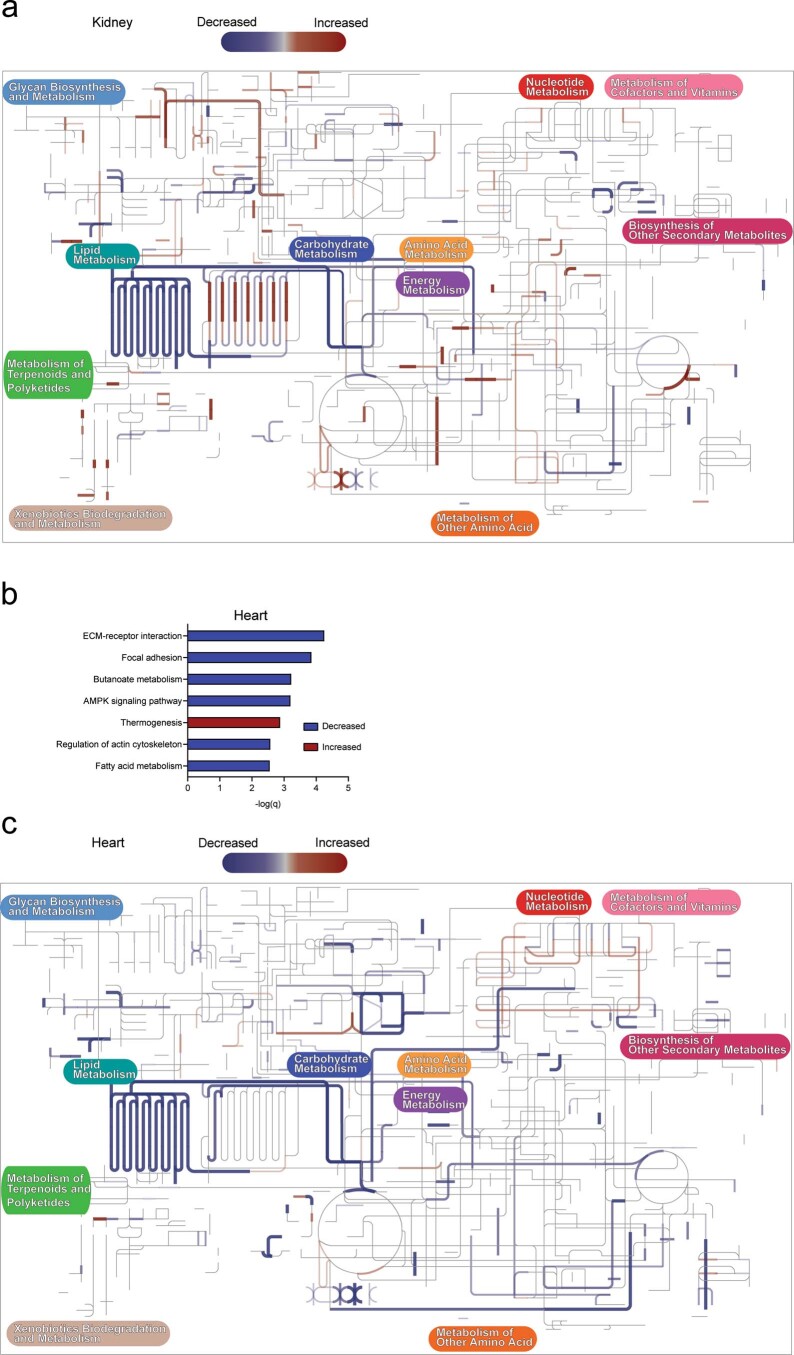
Analysis of GAPDH mutant mice. (**a**) Mapping of differential protein abundance in the kidney on a metabolic pathway chart (iPath). Blue lines highlight metabolic transformations significantly downregulated in GAPDH mutant kidneys. Red lines highlight metabolic transformations significantly upregulated in GAPDH mutant kidneys. The line thickness indicates fold change. (**b**) Gene set enrichment analysis of proteins differentially expressed in the GAPDH mutant heart. Mapping to KEGG (Kyoto Encyclopedia of Genes and Genomes) pathways. Blue bars: gene sets with decreased representation in mutant heart. Red bars: gene sets with increased representation in mutant heart. Based on n = 6 biological replicates. (**c**) Mapping of differential protein abundance in the heart on a metabolic pathway chart (iPath). Blue lines highlight metabolic transformations significantly downregulated in GAPDH mutant kidneys. Red lines highlight metabolic transformations significantly upregulated in GAPDH mutant kidneys. The line thickness indicates fold change. Source data

Finally, to assess if the GAPDH redox switch is also relevant at the level of organismal physiology, we analysed GAPDH(T175A) knock-in mice. These mice do not exhibit any macroscopically visible change in phenotype at young ages (<12 weeks), suggesting that they can compensate for the lack of the redox switch, at least under non-stress homeostatic conditions. However, endogenous production of H_2_O_2_ may be expected to trigger the GAPDH switch at least to some extent in metabolically active tissues or cell types. Erythrocytes are known to be susceptible to oxidative stress, due to haemoglobin autoxidation. As erythrocytes lack mitochondrial metabolism, they depend on the oxPPP for NADPH regeneration and the maintenance of redox homeostasis^25^. Indeed, we observed that erythrocytes from mutant mice exhibit higher endogenous ROS levels than their WT counterparts (Fig. 7a and Extended Data Fig. 6a). The deficiency in erythrocyte redox homeostasis was accompanied by higher erythrocyte counts, increased haematocrit and more megakaryocyte–erythroid progenitors (MEPs) in the bone marrow (Fig. 7b and Extended Data Fig. 6b), suggesting that mutant mice compensate for the impairment of their erythrocytes by increasing erythropoiesis. We then studied kidney and heart, which are metabolically highly active organs. To gain insight into potential adaptive changes caused by the lack of the GAPDH redox switch, we compared protein expression between WT and mutant tissues by deep proteomic analysis. Analysis of the kidney proteome by diaPASEF, followed by DIA-NN analysis^26^, revealed a number of differentially expressed proteins (Fig. 7c). Gene set enrichment analysis indicated significant changes in biosynthetic pathways related to NADPH generation, in particular fatty acid metabolism (Fig. 7d). Metabolic pathway mapping using iPath indicated the downregulation of fatty acid biosynthesis and the upregulation of fatty acid degradation (Extended Data Fig. 7a). An interaction analysis of the most significantly upregulated proteins using String (Fig. 7e) revealed a cluster of enzymes involved in peroxisomal and mitochondrial β-oxidation, including acyl-CoA dehydrogenase (Acadm) and enoyl-CoA hydratase/3-hydroxyacyl-CoA dehydrogenase (Ehhadh) (Fig. 7f), and a cluster of enzymes related to renal blood pressure control, including angiotensin converting enzyme (Ace) and meprin (Mep1a) (Fig. 7g). Furthermore, several antioxidative enzymes were upregulated in the mutant kidney, including mitochondrial thioredoxin (Txn2), peroxiredoxin-5 (Prdx5), glutamate-cysteine ligase (Gclc) and glutaredoxin-5 (Glrx5) (Fig. 7h). Although heart tissues exhibited greater inter-individual variability, gene set enrichment analysis also indicated significant deregulation of fatty acid metabolism, in addition to changes in focal adhesion pathways (Extended Data Fig. 7b). Similar to the kidney, metabolic pathway mapping indicates the suppression of fatty acid biosynthesis in the heart (Extended Data Fig. 7c). In summary, proteomic changes observed in non-stressed knock-in mice suggested that oxidative GAPDH inactivation is relevant in the context of the whole organism, under normal physiological conditions, and across different cell types and organs.

## Discussion

In this study we investigated the relevance of oxidative GAPDH inactivation in mammalian cells. We defined its role in mediating cellular robustness against oxidative stress and explored implications for tumour growth. Finally, we asked if oxidative GAPDH inactivation is relevant in the organismal context and under non-stress conditions. The study provided three major insights.

The first major insight is that reversible oxidation of GAPDH is highly relevant for the triggering of the glycolysis–oxPPP transition and the balancing of NADPH supply in oxidative stress situations. Our study demonstrates for the first time that GAPDH oxidation in mammalian cells not only correlates with, but is actually required for glycolysis–oxPPP rerouting. Although we proposed this mechanism already more than 15 years ago^8^, causality remained to be proven for mammalian cells. In the meantime, it was suggested that PPP ‘reserve flux capacity’ alone may be sufficient to explain increased PPP flux in response to H_2_O_2_, independently of GAPDH oxidation^11^. Eventually, the mechanistic understanding of GAPDH oxidation, together with genome editing, allowed us to inactivate the redox switch without compromising the primary glycolytic function of GAPDH. Our results show that oxidative GAPDH inactivation cooperates with PPP reserve flux capacity to balance the NADPH pool during oxidative stress conditions. Our findings are fully consistent with increased metabolic flux through G6PDH, as triggered by elevated NADP^+^. Indeed, our measurements with the Apollo-NADP^+^ probe (Fig. 2b) confirm that G6PDH is activated under the conditions of metabolic rerouting, and that the metabolic response of G6PDH to changes in the NADPH/NADP^+^ ratio does not require GAPDH oxidation. However, our experiments also show that the activation of G6PDH alone is insufficient to account for PPP activation under conditions of peroxide stress. Hence, our data suggest that both processes, GAPDH inhibition and G6PDH activation, act in concert, as H_2_O_2_ elevation not only leads to GAPDH oxidation, but also to NADPH oxidation. Hence, the allosteric breaks on G6PDH activity are released (by NADP^+^ binding) at the same time when GAPDH inhibition dams up glycolytic flux to make more G6P available.

The second major insight of this study is the (patho-)physiological relevance of the GAPDH redox switch with regard to endogenous oxidant levels. Most previous studies on the glycolysis–oxPPP transition focused on microbes and exogenously applied peroxide stress. Tolerance to environmentally generated peroxides is highly relevant to microbes, such as in microbial communities, in harsh environments or in the context of phagocyte attack. In mammalian cells, however, intracellular sources of H_2_O_2_ are important in numerous physiological and pathophysiological situations, such as changes in oxygen availability, different kinds of metabolic imbalance, loss of matrix attachment, exposure to radiation or toxins, or inflammation. However, it remained unclear whether these situations involve GAPDH oxidation as well as PPP activation, as a physiological response mechanism.

In this study we identified a (patho-)physiologically relevant situation of endogenous peroxide elevation, in which GAPDH oxidation becomes relevant for cell survival, namely loss of substrate attachment. Detached mutant cells, but not WT cells, suffered from elevated peroxide levels and increased cell death, showing that the GAPDH redox switch and its role in activating oxPPP flux, is critical for survival during anchorage-independent growth. While WT and mutant cells behaved similarly in monolayer culture, mutant cells showed increased peroxide stress and cell death during spheroid growth. These results are in line with earlier reports^18,27^, showing that anchorage-independent spheroid growth requires changes in metabolism to suppress an increase in endogenous oxidant levels.

These observations naturally led us to ask about the role of GAPDH oxidation in tumour biology. It has been documented that tumour cells often have to overcome episodes of oxidative stress for survival and spread^27,28^. Tumour cells losing matrix attachment and entering the circulation are observed to experience oxidative stress^17^. Evidence suggests that acutely stressed tumour cells need to rapidly boost reductive capacity to survive for long enough to establish more long-lasting adaptations^18^. Along those lines, the provisioning of antioxidants to mice was found to accelerate disease progression by helping stressed tumour cells to survive^27,29–31^. Taken together, oxidative stress has been suggested to act as a major barrier to tumour progression that malignant cells need to overcome by boosting reductive capacity.

Looking at tumour growth in a mouse xenograft model we found that silencing of the GAPDH redox switch, thus limiting the ability of tumour cells to boost oxPPP flux when needed, substantially limited tumour growth while enhancing endogenous peroxide stress. In other words, the GAPDH redox switch helps tumour cells to activate the oxPPP and to suppress the oxidative stress that is associated with tumour growth. This finding predicted that a pro-oxidative treatment (to enhance oxidative burden on tumour cells) should synergize with the inhibition of the GAPDH redox switch. Indeed, we found that cisplatin as well as radiation treatment, both of which elevated endogenous peroxide levels in tumour cells, strongly enhanced the tumour-suppressive effect of a deficient GAPDH redox switch.

Assuming that many tumours need to overcome conditions leading to peroxide stress, at least locally and/or temporarily, we expect our proof-of-concept tumour experiment to have broader relevance for tumour biology, as it reproduces lack of proper substrate attachment and expansion of the tumour mass into a host environment imposing oxidative stresses (as directly demonstrated by the use of genetically encoded redox probes). However, to assess the relevance of these findings in relation to other tumour entities and host microenvironments, additional work is required.

It has been suggested that robust maintenance of NADPH levels (that is, stress resistance) is often more limiting for tumour growth than are energy levels or biosynthetic precursors^32^. Indeed, the need for stable NAD(P)H levels under stress may contribute to the Warburg effect, that is, the fact that many tumour cells prefer to run on glucose fermentation rather than oxidative respiration, despite the latter producing stoichiometrically more ATP, due to full glucose oxidation. Fermentative glycolytic ATP production, based on high glucose uptake and turnover, arguably is the ideal premise for rapid and dynamic rerouting of glucose flux into the oxPPP, as needed under oxidative stress and triggered by the GAPDH switch.

The glycolysis–oxPPP transition seems to operate as a fast-acting first-line response to oxidative stress and may often be essential to buy sufficient time for stressed cells to establish longer-lasting adaptations. It is well known that normal and tumour cells can use different kinds of long-term adaptions to increase reductive capacity, as there are additional pathways that can serve to regenerate NADPH, for example, the folate pathway^33^, although these are believed to be less responsive and of lower flux than the oxPPP. Hence, future studies should address how some tumour cells manage to adapt to an inactivated GAPDH redox switch.

Finally, the third major insight of this study is that the GAPDH redox switch is relevant in the context of the whole organism, under normal physiological conditions, and across different cell types and organs. The proteome analysis of heart and kidney tissue indicated that mutant mice downregulate fatty acid synthesis while upregulating fatty acid uptake and β-oxidation. Since fatty acid synthesis is a major consumer of NADPH, its downregulation is likely to represent an adaptation to defective oxPPP activation. It is less obvious how increased β-oxidation may contribute to adaptation. Increased supply of acetyl-CoA for the Krebs cycle may enhance NADPH regeneration through either malic enzyme or isocitrate dehydrogenase^34–36^. It is also possible that β-oxidation-derived NADH helps to replenish NADPH through the action of nicotinamide nucleotide transhydrogenase^37^. Taken together, it appears that young adult knock-in mice, despite showing signs of increased oxidative stress (Fig. 7a,h), are overall able to compensate for deficiencies that occur in tissues with naturally elevated endogenous peroxide production. It will therefore be interesting to investigate mice of older age in future experiments. It is also an interesting question if growing tumours adapt to the lack of the GAPDH redox switch by increasing β-oxidation, considering the role of β-oxidation for several cancer types, including pancreatic ductal adenocarcinoma^38^ and prostate cancer^39^.

## Methods

### Cell lines

HAP1 cells were purchased from Horizon Genomics. HAP1-GAPDH(Y314F) cells were generated by CRISPR/Cas9-mediated genome editing using gRNAs TCTAGGTATGACAACGAATT and TTTTCTGAGCCAGCCACCAG. Two clonal cell lines were independently selected. GAPDH(T175A) MEFs were obtained from GAPDH(T175A) knock-in mice. All cell lines were cultured in Iscove’s modified Dulbecco’s medium (IMDM; Life Technologies), supplemented with 10% bovine calf serum (Life Technologies) and 50 units ml^−1^ of penicillin and streptomycin (Life Technologies). All cell lines were regularly tested for mycoplasma contamination, viral infections and cell line identity by multiplex PCR sequencing (Multiplexion).

### Whole genome sequencing and CRISPR off-target site prediction

Fastq files were aligned to the human genome (build hs37d5) using bwa mem (http://bio-bwa.sourceforge.net/bwa.shtml), followed by PCR de-duplication with Picard tools (http://broadinstitute.github.io/picard/index.html). Sequencing statistics were obtained from mosdepth^40^, which indicated an average whole genome coverage of 37X. Somatic variants were identified using Strelka variant caller^41^, and further annotated with the Ensembl Variant Effect Predictor^42^. Variants annotated in dbSNP with minor allele frequency above 1% were filtered. Potential CRISPR off-target sites were computationally predicted using the CRISPRseek Bioconductor package, and checked against identified somatic mutations from whole genome sequencing^43^.

### Stable expression of biosensors in HAP1 cells

HAP1 cells stably expressing roGFP2-Orp1 were generated by retroviral transduction. The pLPCX roGFP2-Orp1 plasmid (addgene #64991) was transfected into 3.5 × 10^6^ Phoenix-AMPHO cells grown in a T75 flask. After 24 h, 12 ml medium was collected and passed through a 0.45 µm filter. Then, 12 ml Retro-X Concentrator (Takara Biosciences #631455) was added, followed by incubation (1 h at 4 °C) and centrifugation (1,500*g*, 45 min at 4 °C). The supernatant was removed and complexes were resuspended in 2.25 ml of IMDM. Then, 8 µg ml^−1^ protamine sulfate (Sigma) was added. The transduction mixture was then added to 4 × 10^5^ HAP1 cells in six-well format. Cells were spinfected (800*g*, 1 h, room temperature (RT)). Forty-eight hours thereafter, cells were selected with puromycin (1 µg ml^−1^). Seventy-two hours thereafter, cells were FACS-sorted for homogeneous GFP fluorescence.

HAP1 cells expressing the Apollo-NADP^+^ probe were generated by lentiviral transduction. The pECFP Cerulean-tagged Apollo-NADP^+^ plasmid (addgene #71800) was used to clone the Apollo-NADP^+^ probe into the LeGO-iT2 vector backbone (addgene #27343) after restriction digest with BamH1 + BsrGI.

(Forward primer: ACACAGGTGTCGTGACGCGGATGGCAGAGCAGGTGGCC, Reverse primer: GCTGGCGGCCGGCCGCTTTACTTTTACTTGTACAGCTCGTCCATGC). A total of 3.5 × 10^6^ HEK293T cells were transfected with pMD2G, psPAX2 and LeGO-iT2_ Apollo-NADP^+^ in a T75 flask. After 24 h, 12 ml medium was collected and passed through a 0.45 µm filter. Then, 12 ml Retro-X Concentrator (Takara Biosciences #631455) was added, followed by incubation (1 h at 4 °C) and centrifugation (1,500*g*, 45 min at 4 °C). The supernatant was removed and complexes were resuspended in 2.25 ml of IMDM. Then, 8 µg ml^−1^ protamine sulfate (Sigma) was added. The transduction mixture was then added to 4 × 10^5^ HAP1 cells in six-well format. Cells were spinfected (800*g*, 1 h, RT). Forty-eight hours thereafter, cells were selected with neomycin (1 µg ml^−1^). Seventy-two hours thereafter, cells were FACS-sorted for homogeneous Cerulean fluorescence.

### GAPDH activity assay

Cell lysate GAPDH activity was measured using the PromoCell GAPDH activity kit (#PK-CA577-K680). In brief, 1 × 10^5^ cells were seeded in 48-well format overnight and treated with 100 µM H_2_O_2_ in a volume of 100 µl for 5 min. Cells were lysed and assayed as described by the manufacturer.

### Relative quantitation of glutathionylated GAPDH

A total of 5 × 10^5^ cells were seeded in six-well format overnight, treated with 100 µM H_2_O_2_ in 1 ml volume for 90 s, incubated with 100 mM *N*-ethylmaleimide (NEM) for 10 min and lysed in RIPA buffer containing 10 mM NEM and Complete Protease Inhibitor Cocktail (Roche #4693124001). Per sample, 15 µg of protein were separated by SDS–PAGE. A gel piece covering the expected molecular weight (37 kDa) was cut out and processed as described^44^. In brief, trypsin digestion was done overnight at 37 °C. The reaction was quenched by addition of 20 µl of 0.1% trifluoroacetic acid (Biosolve) and the supernatant was dried in a vacuum concentrator. Nanoflow liquid chromatography with tandem mass spectrometry (LC–MS/MS) analysis was performed with an Ultimate 3000 liquid chromatography system coupled to an QExactive HF mass spectrometer (Thermo Fisher). Samples were dissolved in 0.1% trifluoroacetic acid, injected to a self-packed analytical column (75 µm × 200 mm; ReproSil Pur 120 C18-AQ; Dr. Maisch GmbH) and eluted with a flow rate of 300 nl min^−1^ in an acetonitrile gradient (3–40%). The mass spectrometer was operated in data-dependent acquisition mode, automatically switching between MS1 and MS2. Collision-induced dissociation MS2 spectra were generated for up to 20 precursors with normalized collision energy of 29%. In some cases, 2 of the 20 precursor values were set to fixed *m*/*z* values of 985.5 and 992.0, to target the doubly charged peptide IISNASCTTNCLAPLAK carrying either two NEM modifications or one NEM modification plus one modification corresponding to the molecular weight of glutathione. Raw data files were processed using Proteome discoverer 2.1 (Thermo Scientific) for peptide identification and quantification. MS2 spectra were searched against the human proteome database (UniprotKB) and the contaminants database (MaxQuant; MPI Martinsried) with the following parameters: Acetyl (Protein N-term), Oxidation (M), NEM (C) and NEM + H_2_O (C) as variable modifications, and trypsin/P as the proteolytic enzyme with up to two missed cleavages was allowed. The maximum false discovery rate for proteins and peptides was 0.01, and a minimum peptide length of seven amino acids was required.

### Detection of GAPDH hyperoxidation

A total of 5 × 10^5^ cells were seeded into six-well plates, incubated overnight, treated with H_2_O_2_ (0–2 mM) in 1 ml volume for 5 min, incubated with 100 mM NEM for 10 min and lysed in RIPA buffer containing 10 mM NEM and Complete Protease Inhibitor Cocktail (Roche #4693124001). Per sample, 15 µg of total protein was run on a reducing gel, transferred onto a PVDF membrane and immunoblotted against GAPDH-SO_2/3_ (1:2,000, Invitrogen #LF-PA0006), GAPDH (1:2,500, abcam #9485), β-Actin (1:10,000, Sigma #A5441) and β-Tubulin (1:1,000, Cell Signaling #2128).

### Metabolic flux analysis

A total of 5 × 10^6^ cells were seeded in 150 mm dish format overnight. The next day, medium was changed to 9 ml Dulbecco’s modified Eagle’s medium containing 5 mM glucose + 10% FCS (base medium) and cells incubated for 2 h. Base medium + 50 mM [1,2-^13^C] glucose and 100/500 µM H_2_O_2_ (labelling medium) was prepared. Finally, 10% labelling medium was added to the cells. All following steps were performed with ice-cold solutions, unless otherwise indicated. Cells were rinsed quickly 2× with PBS, placed on dry ice and quenched with 840 µl H_2_O:MeOH (4:10 v/v), scraped and transferred into a 2 ml Eppendorf tube. Extracts were sonicated 5× for 3 s in ice-cold water. Then, 240 µl of CHCl_3_ (Sigma #650498) was added, vortexed, 240 µl of H_2_O (Carl Roth #A511.1) was added, vortexed and, finally, another 240 µl of CHCl_3_ was added, vortexed and extracts centrifuged for 30 min at 2,200*g* and 4 °C. The aqueous phase was dried down in a vacuum concentrator. Samples were resuspended in 50 µl H_2_O:MeOH (1:1 v/v), centrifuged (5 min, 15,000*g*, 4 °C) and transferred into mass spectrometry vials. LC–MS/MS analysis was performed as previously described^45^. In short, intermediates of glycolysis and the pentose phosphate pathway were resolved on an Agilent 1290 liquid chromatography system, using a HILIC amide column (Waters BEH Amide, 2.1 × 100 mm, 1.7 µm particle size) with acetonitrile (solvent A) and 100 mM aqueous ammonium carbonate (solvent B) at a constant flow rate of 0.3 ml min^−1^, and a column temperature of 35 °C. The gradient started at 30% B and was kept constant for 3 min before a steady increase to 60% B over 4 min. Solvent B was maintained at 60% for 1 min before returning to initial conditions. The column was washed and equilibrated for 2 min, resulting in a total analysis time of 10 min. The flow was coupled to an Agilent 6470 triple quadrupole mass spectrometer operating in tandem MS mode. Isotopologues of sugar phosphates were monitored using the transition from the intact molecule to dihydrogen phosphate (H_2_PO_4_)^−^ at *m*/*z* 97. To assure similar integration for all isotopologues, peaks were integrated with the same defined time segment using Agilent Masshunter software (B.07.01). The recorded signals were corrected for the natural isotopologue distribution using the R package BRAIN^46^. We tested for interferences of signals in samples without the ^13^C-tracer. Due to the substantial contribution of noise in higher-weight isotopologues, we removed signals greater than M + 3, considering their contribution negligible during the short labelling period. NADP^+^ and NADPH were quantified by external calibration using the M + 0 signal.

### Measurement of the ECAR

The extracellular acidification rate (ECAR) was measured on a Seahorse XFe96 (Agilent). A total of 4 × 10^4^ cells were seeded overnight into an Agilent Seahorse 96-well XF Cell Culture Microplate. The XFe96 cartridge was hydrated as described by the manufacturer. The next day, cells were washed with PBS and kept in minimal Dulbecco’s modified Eagle’s medium (Gibco A1443001) supplemented with 2% of FCS for 30 min. Thereafter, the assay was started. After five cycles, glucose was injected to a final concentration of 8 mM, and after another five cycles, H_2_O_2_ was injected to a final concentration of 200 μM. Another eight cycles were recorded thereafter. An ECAR ratio was calculated by dividing the value obtained after addition of H_2_O_2_ (that is, cycle 12) by the value obtained before addition of H_2_O_2_ (that is, cycle 10).

### Measurement of relative NADPH levels

Relative intracellular NADPH levels were determined using an NADPH assay kit (abcam #186031), as described by the manufacturer. Briefly, 1 × 10^5^ cells were seeded overnight in 48-well format. The next day, cells were treated with 100 µM H_2_O_2_ in 100 µl volume for 5 min and NADPH levels determined.

### Fluorescence polarization measurement of the Apollo-NADP^+^ probe

Relative changes in cellular NADP^+^ levels were measured by fluorescence polarization, using the Apollo-NADP^+^ probe^15^. Briefly, 7.5 × 10^4^ HAP1 cells expressing Apollo-NADP^+^ were seeded overnight in 96-well (black wall/clear bottom) format. The next day, fluorescence polarization was measured with a microplate reader (PHERAstar FS, BMG Labtech) equipped with a fluorescence polarization filter (430 nm excitation, 480 nm emission). Then, 100 µM H_2_O_2_ was added in a final volume of 200 µl and the probe response monitored. Polarization values were calculated as *P* = [(*I*_∥_ − *G* × *I*_⊥_)/(*I*_∥_ + *G* × *I*_⊥_)]. The compensation factor (*G*) was set automatically dependent on fluorescence gain settings. Polarization values are expressed as mP values.

### Electrode measurement of extracellular H_2_O_2_

Extracellular H_2_O_2_ levels were recorded with the LAB-TRAX-4 free radical analyser (WPI) using the ISO-HPO-2 electrode. Briefly, 5 × 10^5^ cells were seeded overnight in six-well format. The next day, cells were washed with and assayed in imaging buffer (130 mM NaCl, 50 mM KCl, 1 mM MgCl_2_, 1 mM CaCl_2_ and 20 mM HEPES, with and without 10 mM d-glucose). Then, 150 or 200 µM of H_2_O_2_ was added under mild agitation, to a final volume of 4 ml and the electrode response recorded over time.

### Real-time measurement of cytoplasmic H_2_O_2_ levels

Intracellular H_2_O_2_ levels were measured with the roGFP2-Orp1 probe stably expressed in the cytosol of HAP1 cells as previously described^47^: 6 × 10^4^ HAP1 cells expressing the roGFP2-Orp1 probe were seeded overnight in 96-well (black wall/clear bottom) format. The next day, fluorescence emission at 520 nm was monitored following sequential excitation at 405 nm and 488 nm. Then, 100 µM of H_2_O_2_ was added in a final volume of 200 µl and the probe response was recorded using a microplate reader (PHERAstar FS, BMG Labtech). Probe oxidation is expressed as degree of oxidation (OxD). To obtain values for full oxidation and full reduction, each sample was treated with 20 mM *N*,*N*,*N*′,*N*′-tetramethylazodicarboxamide (diamide) and 100 mM dithiothreitol, respectively. OxD was calculated as

$$\begin{array}{l}{\mathrm{OxD}}\ = \frac{{I_{405}\; \times I_{488}\left( {{\mathrm{red}}} \right) - I_{405}\left( {{\mathrm{red}}} \right)\; \times I_{488}}}{{I_{405}\; \times I_{488}\left( {{\mathrm{red}}} \right) - I_{405}\; \times I_{488}\left( {{\mathrm{ox}}} \right) + I_{405}\left( {{\mathrm{ox}}} \right)\; \times I_{488} - I_{405}\left( {{\mathrm{red}}} \right)\; \times I_{488}}}\end{array}$$

where *I*_405_ and *I*_488_ represent fluorescence intensities after excitation at 405 nm and 488 nm, respectively. *I*_405_(red) and *I*_488_(red) represent the dithiothreitol-treated sample and *I*_405_(ox) and *I*_488_(ox) represent the diamide-treated sample.

### Live cell imaging of cell growth

Cell proliferation was monitored optically with an IncuCyte S3 imaging system. A total of 2,500 cells were seeded in 96-well format in a volume of 200 µl and imaged for 120 h under the conditions specified in the corresponding figure. A measure of cell confluence was calculated with the IncuCyte 2019B software.

### Cell viability measurements

To determine cell viability upon incubation with glucose oxidase (GOX), 1 × 10^5^ cells per well were seeded in 24-well format and allowed to adhere for 4 h. Then, the medium was replaced with medium containing 8 mU ml^−1^ GOX in a final volume of 1 ml, and cells incubated for 24 h. Thereafter, cells were detached, pelleted, resuspended into 200 μl of ViaCount solution (Luminex #4000-0040) and transferred to a clear 96-well plate. A Guava easyCyte HT instrument was used for analysis.

### Colony formation assay

Cells were detached, resuspended in PBS and subjected to cell sorting on a flow cytometer (BD FACSAria III). One-hundred cells were sorted into wells containing 500 µl cell culture medium (24-well format). To prevent attachment, cells were sorted into an ultralow-attachment 24-well plate (Corning #CLS3473). After 15 min or 6 h in the ultralow-attachment plate, cells were transferred to a standard 24-well plate to allow attachment and colony formation. Five days later, medium was removed, and cells were washed once with PBS and stained with Coomassie Blue. Colonies were counted with a benchtop microscope at 5× magnification.

### Measurement of intracellular DCF fluorescence

A total of 10,000 cells were sorted (BD FACSAria III) into a 75 cm^2^ ultralow-attachment cell culture flask (Corning #CLS3814) containing 15 ml cell culture medium. After 15 min or 6 h in the ultralow-attachment flask, cells were collected by centrifugation, resuspended in 1 ml PBS and stained with 5 µM 2′,7′-dichlorodihydrofluorescein diacetate (H2DCFDA; ThermoFisher #D399) for 30 min at 37 °C. Thereafter, cells were washed twice with PBS and analysed by flow cytometry (BD FACSCanto II).

### BrdU incorporation assay

Bromodeoxyuridine (BrdU) incorporation was measured with the Biozol BrdU Cell Proliferation Assay Kit (BIV-K306-200). Briefly, 1 × 10^4^ cells were seeded per well of a 96-well plate. The next day, cells were treated with BrdU as described by the manufacturer and incubated for 4 h including or excluding a 100 µM bolus of H_2_O_2_ in a total volume of 200 µl. Thereafter, cells were fixed, stained with anti-BrdU antibody and horseradish peroxidase conjugated secondary antibody, incubated with 3,3',5,5'-tetramethylbenzidine and absorbance detected as described by the manufacturer.

### Generation of spheroids and live cell imaging

A total of 7,500 HAP1 or MEF cells in 200 µl culture medium were seeded per well into a 96-well ultralow-attachment U-shape plate (ThermoFisher #174925). Cells were sedimented for 10 min at 9*g* and for another 10 min at 12*g*. After 96 h, spheroids were stained with 0.5 µg ml^−1^ Hoechst 33258 and 1 µg ml^−1^ propidium iodide (PI) for 30 min, collected with a P1000 pipette tip, washed in PBS once and transferred to a chambered glass-bottom coverslip (ibidi #80827) containing 200 µl imaging buffer (130 mM NaCl, 50 mM KCl, 1 mM MgCl_2_, 1 mM CaCl_2_, 20 mM HEPES and 10 mM d-glucose) per well. For 2D controls, 1 × 10^4^ HAP1 cells per well were seeded into a chambered glass-bottom coverslip (ibidi #80827) containing 200 µl cell culture medium. The next day, cells were washed in PBS once and kept in 200 µl imaging buffer. Images were captured with a 20× objective on a Zeiss LSM 710 ConfoCor 3 confocal scanning laser microscope (emission 520 nm; excitation 405/488 nm) at 37 °C and 5% CO_2_ using Zeiss Zen 3.0 software. Data analysis and calculation of ratio images was performed with ImageJ 1.52 as described previously^47^.

### Tumour xenograft experiments

All animal experiments received ethical approval and were performed in accordance with the guidelines of the local Governmental Committee for Animal Experimentation (Regierungspräsidium Karlsruhe, Germany, licences 35-9185.81/G-118/18, G-284/18 and DKFZ-213). For in vivo xenograft studies, female NOD SCID gamma mice (8–10 weeks old), recruited from the Center for Preclinical Research, DKFZ, Heidelberg, were kept at a 12 h light–dark cycle with unrestricted Kliba 3307 diet and water. A total of 1 × 10^6^ HAP1 cells were resuspended in 100 µl PBS/growth factor reduced Matrigel (BD) (1:1 v/v) and transplanted subcutaneously into the flank under isoflurane anaesthesia. In case of chemotherapy, the tumour-bearing mice were randomized according to tumour volume immediately before application of the drug. Cisplatin (5 mg kg^−1^ body weight, 10 µl g^−1^ mouse, 0.9% NaCl as vehicle) or vehicle alone were delivered on days 14, 17, 21, 35, 38, 56 and 62 after transplantation. For radiation experiments, the HAP1 cell transplantation was done as described but on the upper hind leg. Reaching a mean tumour volume of about 0.1 cm³, mice were randomized according to tumour volume. Immediately thereafter, radiation was started using an X-ray irradiation system (Faxitron MultiRad 225). Under intraperitoneal anaesthesia with a mix of midazolam/medetomidin (5 mg kg^−1^ and 0.5 mg kg^−1^, 10 µl g^−1^ mouse) and subcutaneous antagonization with a mix of flumazenil/atipamezol in 0.9% NaCl (0.5 mg kg^−1^ and 2.5 mg kg^−1^, 10 µl g^−1^ mouse) four cycles of radiation were applied, each cycle consisting of four consecutive fractions of 2 Gy each per day at intervals of 24 h culminating in 8 Gy per week and 2 weeks of recovery between each cycle. For this purpose, mice were transiently transferred into sterile and heated boxes and positioned with their tumour into the radiation field. Controls were mock treated. Beyond daily health checks, tumour size was measured by caliper in two dimensions two to three times weekly and body weight was taken at least once weekly throughout the experiment. Timepoint of necropsy is as indicated or when mice reached a stop criterion of the German Society of Laboratory Animal Sciences (1.5 cm tumour diameter in one dimension, tumour ulceration, acute bleeding and weight loss ≥20%), here defined as survival. The maximal tumour size/burden was not exceeded in any of the experiments. Tumour tissue samples were snap frozen in liquid nitrogen or processed for FACS analysis (transfer into PBS with NEM, 4 °C), paraffin embedding by fixation in 4% PBS-buffered formaldehyde or embedding in Tissue Tec OCT compound for cryo-conservation. For metabolomics analyses, 2 mg kg^−1^ body weight of [1,2-^13^C]glucose (0.2 mg ml^−1^ in 0.9% NaCl) was injected intravenously 15 min before cervical dislocation. Tumours were explanted and samples snap frozen in liquid nitrogen. Frozen tumour samples were pulverized under a liquid nitrogen atmosphere in a Braun Biosciences Dismembrator and 100 mg powder subjected to metabolite extraction.

### Measurement of the roGFP2-Orp1 redox state in tumours

HAP1 cells or freshly collected xenografted tumour samples expressing the roGFP2-Orp1 probe were used for FACS-based detection of probe oxidation. To conserve the probe redox state, explanted tumours were immediately exposed to 100 mM NEM and then disaggregated into single cells in the presence of 10 mM NEM. Specifically, cells or tumour samples were blocked with 100 mM NEM in PBS for 15 min at 4 °C and then washed with PBS once. Cells were detached and trypsin quenched with cell culture medium. Cells were collected by centrifugation (115*g* for 5 min), resuspended in PBS and transferred to FACS tubes. Tumour samples were disaggregated with trypsin and by sequential pipetting through 10 ml, 5 ml and 2 ml stripettes and P1000 pipettes, collected by centrifugation (115*g*, 5 min), washed 2× in PBS, resuspended in PBS and transferred into FACS tubes. For PI staining, samples were supplemented with 1 µg ml^−1^ PI before analysis. Data were collected using FACSDIVA III on a BD FACSCanto II (10,000–100,000 cells per sample). The fluorescence ratio was calculated for all events. Data analysis was done with FlowJo, Version 10.

### Caspase 3 immunohistochemistry

Caspase 3 activation was detected on tumour sections using an antibody against cleaved Caspase-3 (Cell Signaling #9661). Briefly, tumour tissue samples were embedded into paraffin, sliced, treated with 3% H_2_O_2_ for 10 min, blocked with 5% goat serum, incubated with primary antibody (1:100) overnight, washed, incubated with horseradish peroxidase (HRP) conjugated secondary antibody (Jackson #111-035-144, 1:300), washed, incubated with HRP substrate for 8 min, and counterstained with haematoxylin. Samples were imaged with a Zeiss Axioscan 7 slide scanner, and activated Caspase-3-positive regions were quantitated with ImageJ v1.52.

### Generation of knock-in mice

C57BL/6NTac-GAPDH(T175A) knock-in mice were generated by and purchased from Taconic Biosciences. In brief, superovulated C57BL/6NTac females were mated with C57BL/6NTac males to obtain one cell stage fertilized embryos from oviducts at dpc 0.5. These were placed in M2 medium under mineral oil and microinjected with the gRNA (TGTTTTCTAGACAGCTGTGCA) and recombinant Cas9 protein into the pronucleus. Injected one cell stage embryos were transferred to one of the oviducts of 0.5 dpc pseudopregnant NMRI females. Successful genome editing was verified in F0 founder mice by PCR and restriction analysis. Homozygous C57BL/6NTac-GAPDH(T175A) animals were bred from founder animals. Mice were housed at 55 ± 10% humidity and 22 ± 2 °C ambient temperature and a 12 h light-dark cycle with unrestricted Kliba 3307 diet and water.

#### Analysis of peripheral blood and haematopoiesis

Eight-week-old female C57BL/6NTac-GAPDH(T175A) and C57BL/6NTac mice were used for analysis of peripheral blood and bone marrow haematopoiesis. Briefly, 100 µl of peripheral blood was collected into EDTA-coated tubes via puncture of the vena facialis. Peripheral blood parameters were assessed using a Scil Vet abc Plus for veterinary differential blood count. Bone marrow was isolated as published previously^48^. Briefly, mice were killed by cervical dislocation and soft tissue was removed from hind legs. Long bones were then flushed with 2 ml of ice-cold PBS supplemented with 2% (v/v) FCS/PBS (PAA Laboratories/Sigma Aldrich) using a 1 ml syringe fitted with a 23 gauge needle. The cell suspension was filtered through 40 µm cell strainers (Greiner Bio-One), and the cell number was subsequently assessed using a Scil Vet abc Plus.

### DCF staining of erythrocytes

For the analysis of erythrocyte reactive oxygen species content, 3 µl of peripheral blood was incubated in 10 µM H2DCFDA in Hanks’ Balanced Salt Solution for 30 min at 37 °C. Subsequently, cells were washed with Hanks’ Balanced Salt Solution and incubated in fluorescently labelled monoclonal antibodies directed against cell surface epitopes, as detailed in the next section. The incubation step was 30 min and performed at 4 °C in the dark after which cell surface staining and DCF content were analysed.

### Erythrocytes and MEP staining

Bone marrow cells were stained with fluorescently labelled monoclonal antibodies directed against cell surface epitopes. The incubation step was 30 min and performed at +4 °C in the dark. After the incubation, the bone marrow cell suspension were subjected to erythrocyte lysis by 10 min incubation in 1 ml of ACK lysis buffer (Lonza) at RT. After the lysis step, the cells were washed in 1 ml 2% (v/v) FCS/PBS and resuspended in 200 µl 2% (v/v) FCS/PBS. Cell surface staining and DCF content were analysed by either LSRII or LSRFortessa cytometer (Becton Dickinson) equipped with 350 nm, 405 nm, 488 nm, 561 nm and 641 nm excitation lasers. Manual compensation was performed before the analysis using single-antibody-stained OneComp eBeads (eBioscience). The obtained data were analysed using FlowJo software (Tree star). Erythrocytes were defined as staining negative for CD4 (APC-Cy7, BioLegend, clone GK1.5); CD8 (APC-Cy7, BioLegend, clone 53-6.7); CD11b (APC-Cy7, Invitrogen, clone M1/70); B220 (APC-Cy7, Invitrogen, clone RA3-6B2); Gr1 (APC-Cy7, eBioscience, clone RB6-8C5) and CD41 (PE-Cy7, Invitrogen, clone MWReg30). Additionally, they were defined as staining positive for Ter119 (Pacific Blue, eBioscience, clone TER-119) and CD71 (PE, eBioscience, clone R17217). MEP population was defined as staining negative for a panel of lineage markers (CD4, (Pacific Blue, eBioscience, clone GK1.5); CD8 (Pacific Blue, eBioscience, clone 53-6.7); CD11b (Pacific Blue, eBioscience, clone M1/70); Gr1 (Pacific Blue, eBioscience, clone RB6-8C5); B220 (Pacific Blue, eBioscience, clone RA3-6B2); and Ter119, (Pacific Blue, eBioscience, clone TER-119) as well as for Sca-1 (APC-Cy7, BD Biosciences, clone D7), CD34 (FITC, eBioscience, clone RAM34) and CD16/32 (APC, eBioscience, clone 93). MEPs were additionally defined as staining positive for c-Kit (BV711, BD Biosciences, clone 2B8).

#### Sample preparation for proteomics

For resting proteomic analysis of tissue, male animals were killed by cervical dislocation and organs were immediately removed and snap frozen in liquid nitrogen. Fresh snap-frozen heart tissue was ground before sonication (Braun Dismembrator, cooled to liquid nitrogen temperature, 2,500 r.p.m., 30 s). Kidney tissue was fresh snap frozen without further treatment. Approximately 5 mg of tissue powder was transferred to AFA tubes (Covaris, PN 520292) and filled to 70 µl with RIPA buffer supplemented with 1% SDS. Proteins were extracted and DNA sheared (Covaris LE220Rsc: PIP 350 W, DF 25%, CPB 200, 300 s pulse, two repeats, 20 °C). Protein (25 µg) was used for SP3 protein preparation^49^ on a Biomek i7 workstation with single-step reduction and alkylation. Briefly, 16.6 µl reduction and alkylation buffer (40 mM tris(2-carboxyethyl)phosphine, 160 mM chloroacetamide and 200 mM ammonium bicarbonate (ABC)) was added, and samples were incubated for 5 min at 95 °C and cooled to RT. Proteins were bound to 2.5 µg of paramagnetic beads (1:1 ratio of hydrophilic/hydrophobic beads) by adding acetonitrile to 50%. Samples were washed twice with 80% ethanol and once with 100% acetonitrile, before reconstitution in 35 µl of 100 mM ABC. Digestion was completed overnight at 37 °C using a trypsin/LysC enzyme mix (Promega) at a protein:enzyme ratio of 50:1 (w/w) and stopped with formic acid (0.1%).

#### Liquid chromatography–mass spectrometry

LC–MS analysis was conducted on an EVOSEP One system coupled to a Bruker TimsTOF PRO2 mass spectrometer. Five-hundred nanograms of sample material was loaded on the Evotip according to the manufacturer’s protocol. Liquid chromatography was carried out using the EVOSEP 15 SPD LC method (88 min gradient) with an EV1137 performance column (15 cm × 150 µm, 1.5 µm) at 40 °C, coupled to a 10 µm Zero Dead Volume Captive Spray Emitter (Bruker #1865691). For acquisition in dia-PASEF mode, the acquisition scheme covered the mass range *m*/*z* 400–1,201 and ion mobility range 1/*K*_0_ 0.6–1.6, using 16 frames, with two precursor isolation windows per frame (*m*/*z* 26 window width, *m*/*z* 1.0 overlap between the adjacent windows). Accumulation and ramp times were set to 100 ms.

#### Proteomics data processing

The dia-PASEF raw proteomics data were processed using DIA-NN 1.8 (refs. ^26,50^) in library-free mode by searching against the mouse UniProt reference proteome (UP000000589, date: 18 May 2022). MS1 and MS2 mass accuracy tolerances were fixed to 15 ppm. Match-between-runs was activated, quantification strategy was set to ‘Robust LC (high precision)’ and library generation was set to ‘IDs, RT & IM profiling’. All other settings were kept default.

#### Proteomics data analysis

The RT-normalized precursor intensities were then normalized by applying vwmb normalization^51^. Precursors that were detected in fewer than four samples per group were omitted. To obtain protein quantities, proteotypic peptide intensities were summarized into protein log intensities using the MaxLFQ method^52^ implemented in the iq R package^53^. Statistical analysis of proteomics data was carried out in R 4.1 using the limma R package^54^, modelling protein abundance depending on genotype. False discovery rate correction^55^ was applied to the resulting *P* values, and a fold-change threshold for calling differentially expressed genes was set to 1.25. The fold changes estimated by limma were mapped to the mouse metabolic pathway map using iPath3 (ref. ^56^). Kyoto Encyclopedia of Genes and Genomes (KEGG)^57^ pathway enrichment analysis was carried out using the clusterProfiler R package^58^.

### Statistics

Significance was always determined using the unpaired two-tailed Student’s *t*-test. Significance levels are indicated as *P < 0.05, **P < 0.01, ***P < 0.001, ****P < 0.0001; NS, non-significant. Statistical calculations were performed using GraphPad Prism 8.4.

### Reporting summary

Further information on research design is available in the Nature Portfolio Reporting Summary linked to this article.

## Supplementary information

Reporting Summary

## Data Availability

Data generated and analysed in this study are included in this article. The mass spectrometry proteomics data have been deposited to the ProteomeXchange Consortium via the PRIDE partner repository with the dataset identifier PXD040620. Source data are provided with this paper.

## Source data

Source Data Fig. 1 Numerical data and unprocessed blots.
Source Data Fig. 2 Numerical data.
Source Data Fig. 3 Numerical data.
Source Data Fig. 4 Numerical data.
Source Data Fig. 5 Numerical data.
Source Data Fig. 6 Numerical data.
Source Data Fig. 7 Numerical data.
Source Data Extended Data Fig. 1 Numerical data and unprocessed blots.
Source Data Extended Data Fig. 2 Numerical data and unprocessed blots.
Source Data Extended Data Fig. 3 Numerical data and unprocessed blots.
Source Data Extended Data Fig. 4 Numerical data.
Source Data Extended Data Fig. 5 Numerical data.
Source Data Extended Data Fig. 7 Numerical data.

## References

[1] Shenton D, Grant CM (2003). Protein S-thiolation targets glycolysis and protein synthesis in response to oxidative stress in the yeast *Saccharomyces cerevisiae*. Biochem. J..

[2] Baty JW, Hampton MB, Winterbourn CC (2005). Proteomic detection of hydrogen peroxide-sensitive thiol proteins in Jurkat cells. Biochem. J..

[3] van der Reest J, Lilla S, Zheng L, Zanivan S, Gottlieb E (2018). Proteome-wide analysis of cysteine oxidation reveals metabolic sensitivity to redox stress. Nat. Commun..

[4] Anastasiou D (2011). Inhibition of pyruvate kinase M2 by reactive oxygen species contributes to cellular antioxidant responses. Science.

[5] Peralta D (2015). A proton relay enhances H_2_O_2_ sensitivity of GAPDH to facilitate metabolic adaptation. Nat. Chem. Biol..

[6] Zaffagnini M, Fermani S, Costa A, Lemaire SD, Trost P (2013). Plant cytoplasmic GAPDH: redox post-translational modifications and moonlighting properties. Front. Plant Sci..

[7] Little C, O’Brien PJ (1969). Mechanism of peroxide-inactivation of the sulphydryl enzyme glyceraldehyde-3-phosphate dehydrogenase. Eur. J. Biochem..

[8] Ralser M (2007). Dynamic rerouting of the carbohydrate flux is key to counteracting oxidative stress. J. Biol..

[9] Ralser M (2009). Metabolic reconfiguration precedes transcriptional regulation in the antioxidant response. Nat. Biotechnol..

[10] Kuehne A (2015). Acute activation of oxidative pentose phosphate pathway as first-line response to oxidative stress in human skin cells. Mol. Cell.

[11] Christodoulou D (2018). Reserve flux capacity in the pentose phosphate pathway enables escherichia coli’s rapid response to oxidative stress. Cell Syst..

[12] Christodoulou D (2019). Reserve flux capacity in the pentose phosphate pathway by NADPH binding is conserved across kingdoms. iScience.

[13] Lee C (2004). Redox regulation of OxyR requires specific disulfide bond formation involving a rapid kinetic reaction path. Nat. Struct. Mol. Biol..

[14] Jang C, Chen L, Rabinowitz JD (2018). Metabolomics and isotope tracing. Cell.

[15] Cameron WD (2016). Apollo-NADP^+^: a spectrally tunable family of genetically encoded sensors for NADP^+^. Nat. Methods.

[16] Lange K, Proft ER (1970). Inhibition of the 6-phosphogluconate dehydrogenase in the rat kidney by 6-aminonicotinamide. Naunyn Schmiedebergs Arch. Pharmakol..

[17] Schafer ZT (2009). Antioxidant and oncogene rescue of metabolic defects caused by loss of matrix attachment. Nature.

[18] Jiang L (2016). Reductive carboxylation supports redox homeostasis during anchorage-independent growth. Nature.

[19] Gutscher M (2008). Real-time imaging of the intracellular glutathione redox potential. Nat. Methods.

[20] Pereira PMR (2017). Cancer cell spheroids are a better screen for the photodynamic efficiency of glycosylated photosensitizers. PLoS ONE.

[21] Hole PS (2010). Ras-induced reactive oxygen species promote growth factor-independent proliferation in human CD34^+^ hematopoietic progenitor cells. Blood.

[22] Jackson AL, Loeb LA (2001). The contribution of endogenous sources of DNA damage to the multiple mutations in cancer. Mutat. Res..

[23] Conklin KA (2004). Chemotherapy-associated oxidative stress: impact on chemotherapeutic effectiveness. Integr. Cancer Ther..

[24] Marullo R (2013). Cisplatin induces a mitochondrial-ROS response that contributes to cytotoxicity depending on mitochondrial redox status and bioenergetic functions. PLoS ONE.

[25] Szeinberg A, Marks PA (1961). Substances stimulating glucose catabolism by the oxidative reactions of the pentose phosphate pathway in human erythrocytes. J. Clin. Invest..

[26] Demichev V (2022). dia-PASEF data analysis using FragPipe and DIA-NN for deep proteomics of low sample amounts. Nat. Commun..

[27] Piskounova E (2015). Oxidative stress inhibits distant metastasis by human melanoma cells. Nature.

[28] Gill JG, Piskounova E, Morrison SJ (2016). Cancer, oxidative stress, and metastasis. Cold Spring Harb. Symp. Quant. Biol..

[29] Le Gal K (2015). Antioxidants can increase melanoma metastasis in mice. Sci. Transl. Med..

[30] Sayin VI (2014). Antioxidants accelerate lung cancer progression in mice. Sci. Transl. Med..

[31] Wiel C (2019). BACH1 stabilization by antioxidants stimulates lung cancer metastasis. Cell.

[32] Gruning NM, Ralser M (2011). Cancer: sacrifice for survival. Nature.

[33] Fan J (2014). Quantitative flux analysis reveals folate-dependent NADPH production. Nature.

[34] Kalucka J (2018). Quiescent endothelial cells upregulate fatty acid beta-oxidation for vasculoprotection via redox homeostasis. Cell Metab..

[35] Pike LS, Smift AL, Croteau NJ, Ferrick DA, Wu M (2011). Inhibition of fatty acid oxidation by etomoxir impairs NADPH production and increases reactive oxygen species resulting in ATP depletion and cell death in human glioblastoma cells. Biochim. Biophys. Acta.

[36] Jeon SM, Chandel NS, Hay N (2012). AMPK regulates NADPH homeostasis to promote tumour cell survival during energy stress. Nature.

[37] Smith CD, Schmidt CA, Lin CT, Fisher-Wellman KH, Neufer PD (2020). Flux through mitochondrial redox circuits linked to nicotinamide nucleotide transhydrogenase generates counterbalance changes in energy expenditure. J. Biol. Chem..

[38] Khasawneh J (2009). Inflammation and mitochondrial fatty acid beta-oxidation link obesity to early tumor promotion. Proc. Natl Acad. Sci. USA.

[39] Liu Y (2006). Fatty acid oxidation is a dominant bioenergetic pathway in prostate cancer. Prostate Cancer Prostatic Dis..

[40] Pedersen BS, Quinlan AR (2018). Mosdepth: quick coverage calculation for genomes and exomes. Bioinformatics.

[41] Kim S (2018). Strelka2: fast and accurate calling of germline and somatic variants. Nat. Methods.

[42] McLaren W (2016). The Ensembl variant effect predictor. Genome Biol..

[43] Zhu LJ, Holmes BR, Aronin N, Brodsky MH (2014). CRISPRseek: a bioconductor package to identify target-specific guide RNAs for CRISPR–Cas9 genome-editing systems. PLoS ONE.

[44] Fecher-Trost C (2013). The in vivo TRPV6 protein starts at a non-AUG triplet, decoded as methionine, upstream of canonical initiation at AUG. J. Biol. Chem..

[45] Kamrad S (2020). Pyruvate kinase variant of fission yeast tunes carbon metabolism, cell regulation, growth and stress resistance. Mol. Syst. Biol..

[46] Dittwald P, Claesen J, Burzykowski T, Valkenborg D, Gambin A (2013). BRAIN: a universal tool for high-throughput calculations of the isotopic distribution for mass spectrometry. Anal. Chem..

[47] Morgan B, Sobotta MC, Dick TP (2011). Measuring EGSH and H2O2 with roGFP2-based redox probes. Free Radic. Biol. Med..

[48] Walter D (2015). Exit from dormancy provokes DNA-damage-induced attrition in haematopoietic stem cells. Nature.

[49] Muller T (2020). Automated sample preparation with SP3 for low-input clinical proteomics. Mol. Syst. Biol..

[50] Demichev V, Messner CB, Vernardis SI, Lilley KS, Ralser M (2020). DIA-NN: neural networks and interference correction enable deep proteome coverage in high throughput. Nat. Methods.

[51] Koopmans F, Li KW, Klaassen RV, Smit AB (2023). MS-DAP platform for downstream data analysis of label-free proteomics uncovers optimal workflows in benchmark data sets and increased sensitivity in analysis of Alzheimer’s biomarker data. J. Proteome Res.

[52] Cox J (2014). Accurate proteome-wide label-free quantification by delayed normalization and maximal peptide ratio extraction, termed MaxLFQ. Mol. Cell Proteom..

[53] Pham TV, Henneman AA, Jimenez CR (2020). iq: an R package to estimate relative protein abundances from ion quantification in DIA-MS-based proteomics. Bioinformatics.

[54] Ritchie ME (2015). limma powers differential expression analyses for RNA-sequencing and microarray studies. Nucleic Acids Res..

[55] Benjamini Y, Hochberg Y (1995). Controlling the false discovery rate: a practical and powerful approach to multiple testing. J. R. Stat. Soc. Ser. B.

[56] Darzi Y, Letunic I, Bork P, Yamada T (2018). iPath3.0: interactive pathways explorer v3. Nucleic Acids Res..

[57] Kanehisa M, Goto S (2000). KEGG: Kyoto Encyclopedia of Genes and Genomes. Nucleic Acids Res..

[58] Wu T (2021). clusterProfiler 4.0: a universal enrichment tool for interpreting omics data. Innovation.

