# Supplementary material for: The GAPDH redox switch safeguards reductive capacity and enables survival of stressed tumour cells
Source: Nat Metab. 2023 Apr 6;5(4):660–76. doi: 10.1038/s42255-023-00781-3 (PMC10132988; doi:10.1038/s42255-023-00781-3)
Supplement: Supplementary file 1 — Reporting Summary [file 42255_2023_781_MOESM1_ESM.pdf]

nature portfolio

Corresponding author(s): Tobias P. Dick

Last updated by author(s): Feb 28, 2023

Reporting Summary

Nature Portfolio wishes to improve the reproducibility of the work that we publish. This form provides structure for consistency and transparency in reporting. For further information on Nature Portfolio policies, see our Editorial Policies and the Editorial Policy Checklist.

Statistics

For all statistical analyses, confirm that the following items are present in the figure legend, table legend, main text, or Methods section.

n/a	Confirmed	
☐	☒	The exact sample size (*n*) for each experimental group/condition, given as a discrete number and unit of measurement
☒	☐	A statement on whether measurements were taken from distinct samples or whether the same sample was measured repeatedly
☐	☒	The statistical test(s) used AND whether they are one- or two-sided<br>*Only common tests should be described solely by name; describe more complex techniques in the Methods section.*
☒	☐	A description of all covariates tested
☒	☐	A description of any assumptions or corrections, such as tests of normality and adjustment for multiple comparisons
☐	☒	A full description of the statistical parameters including central tendency (e.g. means) or other basic estimates (e.g. regression coefficient) AND variation (e.g. standard deviation) or associated estimates of uncertainty (e.g. confidence intervals)
☐	☒	For null hypothesis testing, the test statistic (e.g. *F*, *t*, *r*) with confidence intervals, effect sizes, degrees of freedom and *P* value noted<br>*Give P values as exact values whenever suitable.*
☒	☐	For Bayesian analysis, information on the choice of priors and Markov chain Monte Carlo settings
☒	☐	For hierarchical and complex designs, identification of the appropriate level for tests and full reporting of outcomes
☒	☐	Estimates of effect sizes (e.g. Cohen's *d*, Pearson's *r*), indicating how they were calculated

Our web collection on statistics for biologists contains articles on many of the points above.

Software and code

Policy information about availability of computer code

Data collection	Zeiss Zen 3.0<br>Proteome discoverer 2.1<br>Agilent Masshunter B.07.01<br>MaxQuant 1.5.6.0<br>FACSDiva III
Data analysis	FlowJo 10<br>ImageJ 1.52<br>R 4.1<br>DIA-NN 1.8<br>IncuCyte 2019B<br>Picard Tools v 2.27.1<br>Microsoft Excel 2016<br>GraphPad Prism 8.4

For manuscripts utilizing custom algorithms or software that are central to the research but not yet described in published literature, software must be made available to editors and reviewers. We strongly encourage code deposition in a community repository (e.g. GitHub). See the Nature Portfolio guidelines for submitting code & software for further information.

Data

Policy information about availability of data

All manuscripts must include a data availability statement. This statement should provide the following information, where applicable:

- Accession codes, unique identifiers, or web links for publicly available datasets
- A description of any restrictions on data availability
- For clinical datasets or third party data, please ensure that the statement adheres to our policy

Data generated and analyzed in this study are included in this article. Source data are provided with this paper. The mass spectrometry proteomics data have been deposited to the ProteomeXchange Consortium via the PRIDE partner repository with the dataset identifier PXD040620.

Human research participants

Policy information about studies involving human research participants and Sex and Gender in Research.

Reporting on sex and gender	N/A
Population characteristics	N/A
Recruitment	N/A
Ethics oversight	N/A

Note that full information on the approval of the study protocol must also be provided in the manuscript.

Field-specific reporting

Please select the one below that is the best fit for your research. If you are not sure, read the appropriate sections before making your selection.

☒ Life sciences ☐ Behavioural & social sciences ☐ Ecological, evolutionary & environmental sciences

For a reference copy of the document with all sections, see nature.com/documents/nr-reporting-summary-flat.pdf

Life sciences study design

All studies must disclose on these points even when the disclosure is negative.

Sample size	Sample sizes and cell number for cell culture experiments were selected according to experience and current conventions in life science. Experiments were repeated on different days using cells of different passage number. All experiments (excluding animals experiments) utilize at least 3 technical replicates. Xenograft experiments were performed with a minimum of 5 animals per group, except for cisplatin and ionizing radiation experiments, for which biometric calculations were performed (using R 3.4.0) to aim for statistical significance against untreated wt animals, assuming 25% and 40% reduction in tumor volume for treated wt and mutant animals, respectively. This allowed for the utilization of the statistical minimum of animals to be used in the experiment to satisfy scientific and ethical standards of the mouse protocols.
Data exclusions	In xenograft experiments, animals lacking tumor formation were excluded from analysis (in total 2 out of >100 animals). No other data was excluded.
Replication	All attempts at replication were successful. Cell culture and in vitro experiments were performed with at least 3 biological replicates. In Fig. 3, one representative experiment (out of 3 biological replicates with 5 technical replicates each) is shown. Cell culture experiments were repeated by at least one more author (two authors performed cell culture experiments.) Biochemical assays were also repeated at least 3 times.
Randomization	LC/MS samples were measured in a randomized order on the LC queue. Cage mate animals were randomly assigned for treatment type (chemotherapeutic, radiation, or sham) for both xenograft and proteomics experiments. To minimize variation, animals of the roughly the same age (within 30 days of total age difference) were used and terminal experiments were performed at the same time of day to limit circadian effects on metabolism and gene expression. Samples for biochemical assays and cell culture were not randomized because they were defined groups (by genotype.)
Blinding	For proteomic analysis of resting kidney and heart tissue, researchers were blinded to the genotype and treatment of the animals until after data collection and processing was complete. At this point, researchers were unblinded to create data comparisons based on genotype. Similarly for xenograft experiments, animal weight and tumor size data were collected by technicians who did not know the genotype of the animals and data collection was performed blinded. Researchers were unblinded for compilation and analysis of data. Blinding was not carried out in cell culture experiments because there were defined groups and blinding was not necessary (by genotype).

Reporting for specific materials, systems and methods

We require information from authors about some types of materials, experimental systems and methods used in many studies. Here, indicate whether each material, system or method listed is relevant to your study. If you are not sure if a list item applies to your research, read the appropriate section before selecting a response.

Materials & experimental systems

n/a	Involved in the study
☐	☒ Antibodies
☐	☒ Eukaryotic cell lines
☒	☐ Palaeontology and archaeology
☐	☒ Animals and other organisms
☒	☐ Clinical data
☒	☐ Dual use research of concern

Methods

n/a	Involved in the study
☒	☐ ChIP-seq
☐	☒ Flow cytometry
☒	☐ MRI-based neuroimaging

Antibodies

Antibodies used

Anti GAPDH (1:2,500 abcam #9485)
Anti GAPDH-SO2/3 (1:2,000 Invitrogen #LF-PA0006 Lot Number: QD2008551)
Anti ß-Actin (1:10,000 sigma #A5441)
Anti ß-Tubulin (1:1,000 cell signaling #2128).
Anti BrdU (1:100, Merck QIA58, HTS01)

Validation

All antibodies used in this study are commercially available with manufacturer provided certificates of analysis and have been extensively referenced in literature and were used without further validation.
GAPDH (abcam #9485) - According to manufacturer's website (last accessed 2022/11/03), product has 2293 citations and is valid for western blot, immunocytochemistry and immunohistochemistry against human and mouse GAPDH - https://www.abcam.com/products/primary-antibodies/gapdh-antibody-loading-control-ab9485.html
GAPDH-SO2/3 (Invitrogen #LF-PA0006 Lot Number: QD2008551) - Manufacturer lists western blot and a positive reactivity with human GAPDH-SO2/3 on Lot Certificate of Analysis (last accessed 22/11/03) - https://www.thermofisher.com/antibody/product/GAPDH-Antibody-Polyclonal/LF-PA0006
ß-Actin (sigma #A5441) - According to manufacturer's website (last accessed 2022/11/03), product has 9405 citations and is valid for western blot and immunohistochemistry against pig, Hirudo medicinalis, bovine, rat, canine, feline, human, rabbit, carp, mouse, guinea pig, chicken, and sheep ß-Actin - https://www.sigmaaldrich.com/DE/en/product/sigma/a5441
ß-Tubulin (cell signaling #2128) - Manufacturer's product data sheet (last accessed 2022/11/03) indicates product is suitable for WB, IHC-P, IF-IC, FC-FP and is reactive to human, mouse, rat, monkey, zebrafish and bovine ß-Tubulin and has be used in 693 publications - https://www.cellsignal.com/products/primary-antibodies/b-tubulin-9f3-rabbit-mab/2128
anti-BRDU (Merck QIA58, HTS01) - part of a kit that has been validated (last accessed 2023/02/23) - https://www.merckmillipore.com/DE/de/product/BrdU-Cell-Proliferation-Assay,EMD_BIO-QIA58

Eukaryotic cell lines

Policy information about cell lines and Sex and Gender in Research

Cell line source(s)

Human HAP1 cells were obtained from Horizon Genomics. HAP1_roGFP2-Orp1, HAP1_Y314F_roGFP2-Orp1, HAP1_Apollo-NADP+, HAP1_Y314F_Apollo-NADP+, MEF and MEF_T175A were generated in this study. HEK293T cells were obtained from ATCC.

Authentication

HAP1 cell lines were genome sequenced. Targeted mutations in MEF cell lines were confirmed by PCR . HEK293T cells were validated by multiplex PCR sequencing (Multiplexion, Heidelberg).

Mycoplasma contamination

All cell lines were tested regularly for mycoplasma contamination, viral infections and cell line identity by multiplex PCR sequencing (Multiplexion, Heidelberg). All cell lines tested negative for these contaminations.

Commonly misidentified lines
(See ICLAC register)

No commonly misidentified cell line was used in this study.

Animals and other research organisms

Policy information about studies involving animals; ARRIVE guidelines recommended for reporting animal research, and Sex and Gender in Research

Laboratory animals

Mus musculus: NSG mice, female, 6-8 weeks of age were housed at 55±10% humidity and 22±2 C ambient temperature and a 12 h light-dark cycle with unrestricted Kliba 3307 diet and water. C57BL/6NTac mice, 10-12 weeks of age, obtained from Taconic Biosciences (Köln, Germany) were housed with 3-5 littermates of the same sex at 55±10% humidity and 22±2 C ambient temperature and a 12 h light-dark cycle with unrestricted Kliba 3307 diet and water.

Wild animals	No wild animals were used in this study.
Reporting on sex	Female animals were used for xenograft experiments to eliminate sex as a possible variable and to minimize the number of animals used. To show that effects of the GAPDH_T175A mutation are not sex specific, proteomics analysis was performed on male animals.
Field-collected samples	No field-collected samples were used in this study.
Ethics oversight	All animal experiments were approved by a governmental ethics committee for animal welfare (Regierungspäsidium Karlsruhe, Germany, under licence no. G118-18). Animal welfare was checked on a daily bases by animal care takers and on demand by animal facility veterinarians. In addition, experimenters checked for humane endpoints according to the licensed frequency.

Note that full information on the approval of the study protocol must also be provided in the manuscript.

Flow Cytometry

Plots

Confirm that:

☒ The axis labels state the marker and fluorochrome used (e.g. CD4-FITC).

☒ The axis scales are clearly visible. Include numbers along axes only for bottom left plot of group (a 'group' is an analysis of identical markers).

☒ All plots are contour plots with outliers or pseudocolor plots.

☒ A numerical value for number of cells or percentage (with statistics) is provided.

Methodology

Sample preparation	Tissues from animals were collected immediately after terminal cardiac puncture (done under isoflurane anesthesia) and snap frozen in liquid nitrogen or fixed in neutral buffered formalin. HAP1 cells were grown from thawed aliquots, stored in liquid nitrogen, from an initial, low-passage experiment. MEF cells were derived from the whole uteri of timed-pregnancy females.
Instrument	For cell surface staining of erythrocytes and megakaryocyte-erythroid precursors, LSRII and LSRFortessa cytometers (Becton Dickinson) were used. For measurement of DCF or roGFP2 fluorescence in cell culture cells a BD FACSCanto II (Becton Dickinson) cytometer was used.
Software	Data was collected with BD FACSDiva 8 and analyzed using FlowJo 10. Statistics and figures were prepared with GraphPad Prism 8.4.
Cell population abundance	No cell sorting was applied
Gating strategy	The gating strategy for erythrocytes from peripheral blood and for megakaryocyte-erythroid precursor cells from bone marrow is shown in Extended Data Figure 6. For DCF/roGFP2 measurements in HAP1/MEFs no gating strategy was applied.

☒ Tick this box to confirm that a figure exemplifying the gating strategy is provided in the Supplementary Information.
